# Decision-making and dynamics of eye movements in volleyball experts

**DOI:** 10.1038/s41598-020-74487-x

**Published:** 2020-10-14

**Authors:** Daniel Fortin-Guichard, Vincent Laflamme, Anne-Sophie Julien, Christiane Trottier, Simon Grondin

**Affiliations:** 1 School of Psychology, Université Laval, Quebec, Canada; 2 Department of Mathematics and Statistics, Université Laval, Quebec, Canada; 3 Department of Physical Education, Université Laval, Quebec, Canada

**Keywords:** Human behaviour, Decision

## Abstract

Key decision-makers among experts in a given field can sometimes be identified based on their role and responsibilities. The aim of the study is to compare perceptual-cognitive skills of experts with decisional responsibilities (setters in volleyball) with that of other volleyball experts. Eighty-two participants (26 setters, 36 other players and 20 controls) viewed 50 volleyball video sequences. Sequences stopped 120 ms before ball contact and participants, whose eye movements were recorded, had to predict the ball direction. Generalized Estimating Equations analysis revealed that setters and controls made more but shorter fixations than other players. However, both expert groups made better predictions than controls. Dynamics analyses of eye movements over time show that, right before ball contact, opposing players’ upper body is a most relevant attentional cue in all game situations. Results are discussed in terms of decision-making responsibilities to identify key decision-makers in volleyball and in general. They point towards specific perceptual-cognitive abilities found in setters and support the idea that they constitute a subgroup of experts, but that they are not “better” than other players in anticipating the game.

## Introduction

In sports science, researchers often try to isolate characteristics of subgroups of athletes in order to maximize the comprehension of expert performance^1,2^. In this vein, knowing the characteristics needed for a position in team sports can inform coaches whether a player would be better suited for one position or another. For example, attackers, defenders and goalkeepers in ice hockey differ on anthropometric, strength and aerobic characteristics^2,3^. From an expertise perspective, volleyball is quite interesting: it is one of the sports where specialization in a specific position is the most marked^4^. However, to the authors’ knowledge, an important component of expert performance in volleyball, namely context-specific perceptual-cognitive skills, have never been studied as a function of players’ position. Informing on different positions’ specific perceptual-cognitive abilities could serve as a first empirical step towards the identification of key decision-makers (in volleyball, and in general) based on their role and responsibilities. This could hold even more true for setters in volley-ball, considering that a main ability expected from an athlete playing at this position is decision-making.

Perceptual-cognitive skills are often studied using the “experts-novices” paradigm in order to identify how life-long training can influence one’s ability to “read the game”^5^. Perceptual-cognitive skills in sports refer to abilities like identifying relevant visual cues within a context-specific scene, gather as much information as possible from those cues and accurately anticipate opponents’ actions^6^. Tracking the eye movements of athletes as well as testing their anticipation efficacy (e.g., predicting the follow-up action after temporal occlusion of video sequences^7^), are frequently used to measure these skills. In eye-tracking, ocular fixation can correspond to information processing^8^, but it is not always the case, as research in peripheral vision showed that what athletes fixate does not always correspond to the direction of their attention^9^. Indeed, in some cases, expert athletes can fixate on functional spaces between areas of interest (AOI)^10^. Importantly, eye-tracking and temporal occlusion come with their share of limitations. For example, in eye-tracking, researchers need to deduce peripheral information processing rather than having a clear idea of where attention is directed^9^. As for temporal occlusion, it has been extensively questioned in relation to its usefulness for transferring the findings from the laboratory to the field^11^. However, these measures still give researchers an insight on athletes’ fundamental perceptual-cognitive processes and provide stepping stones towards a full understanding on how athletes “read the game”.

Research from the experts-novices paradigm in sports indicates that fixation number and duration do vary according to expertise, but also as a function of the task at hand and of the response mode. In other words, there is more than a unique attentional pattern to analyze when novices and expert athletes’ gaze behavior have to be compared. The first attentional pattern found using eye-tracking showed that when athletes watch brief video sequences of their sport of expertise, they differ from novices as they pay attention to less visual cues, but fixate for a longer period^6,12,13^. They also anticipate better what is coming next^14^. These seminal studies already showed that experts know where to look at to better anticipate actions, without having to scan avidly through the scene, as novices would do. However, more recent research showed that the more stimuli and the more time an athlete has to anticipate his opponent’s action, the more ocular fixations will be performed; such a pattern is not observed with less skilled participants. The fixations of athletes are most often placed on relevant AOIs^15,16^. With regards to response-mode representativeness, it has been shown that when an athlete has to perform the actual task that is usually required in a game, their attentional patterns are not the same as the ones used when they respond verbally or by moving a joystick, or when they only mimic a sport action^17,18^. For example, Dicks and his colleagues^18^ observed that soccer goalkeepers fixate equally at the penalty kick taker’s movements and at the ball trajectory when they actually try to stop the ball, whereas they mostly looked at the penalty taker’s movements in all other response conditions.

Results from studies in perceptual-cognitive skills in volleyball do not escape this trend, as they also diverge according to task representativeness and response mode. For example, when asked to watch video sequences on a normal-sized computer screen, experts tend to focus on the opposing setter’s upper body to anticipate his action^19^. However, when they are facing near life-size video sequences and are asked to imitate the actions required by a blocker, they use an attentional pivot between multiple targets to anticipate the opposing setter’s action^20^. In addition to diverging results, research in this field include methodological limitations preventing full generalization of results and inviting for caution in their interpretation. For example, in some of these studies, video sequences of practice sessions rather than actual games were presented (i.e., the volleyball coach tosses a ball to the setter^19,21^). The most important critique to put forward regards the lack of sophistication with respect to attentional pattern analysis (i.e., the order in which AOIs are fixated). Button, Dicks, Haines, Barker and Davids^22^ observed that attentional pattern in sports (soccer in that case) can be studied using time-series analysis, presenting a continuous and fluid evolution of attentional pattern. This novel approach informs not only on attentional cues useful in anticipation and decision-making, but also in which order those cues should be attended to. Vansteenkiste and colleagues^20^ adopted this approach in volleyball by showing near life-size video sequences to novices, intermediates and experts. These sequences included a reception, a set and an attack. Sequences were separated in 36 segments, and results show a similar gaze behavior across groups: gaze started between relevant cues as participants were required to fixate the center of the screen at onset, then switched to the receiver, then to the ball after reception. Before the ball reached its highest point, participants started fixating the setter. Once the ball got close to the setter, the number of inter-event fixations increased. Finally, after the setter performed a set, the hitter was the target the most looked at (similar results, yet more detailed, were obtained from verbal report of beach volley-ball players, where a typical gaze behaviour was reception—set—hitter’s approach—hitter’s body direction—hitter’s position relative to the ball—shoulder—arm—elbow—wrist/hand—ball^23^). The main difference between groups in Vansteenkiste and colleagues’^20^ study was located in the duration of inter-event fixations, with experts being faster to reach targets after an inter-event fixation. They probably used their peripheral vision more efficiently to interpret the global scene more quickly. It is noteworthy that separating the three types of ball contact in only 36 segments might have resulted in a lack of details in the analyses. In sum, it appears that studies showing more ecological video sequences and presenting more appropriate analyses of attentional patterns are needed.

### Comparison of perceptual-cognitive skills among experts

In the last two decades, an observable trend in sports psychology^10,24,25^, invite researchers to clarify the notion of perceptual-cognitive expertise by comparing experts among themselves. These studies aim at isolating the most subtle factors involved in expert performance to better inform coaches and athletes on how to train. These comparisons also inform on key factors promoting decision-making and attentional expertise. It seems that expert athletes can be distinguished amongst themselves based on their performance^25^, the number of years of experience^24^ and the evaluation of coaches with regard to their anticipation and decision-making abilities^10^. These studies illustrate that among experts practicing the same sport, it is possible that distinctions exist regarding their perceptual-cognitive processes when facing sports situations. Knowing that, could specialization at a specific position distinguish the experts from each other?

Research comparing expert athletes based on their playing position in team sports yielded diverging results. For example, in-fielders, out-fielders and pitchers in women’s softball differ with regard to anticipation skills when facing near life-size video sequences of a batter from the perspective of an in-fielder^26^. However, in Australian football, players did not differ with regards to playing position on their decision-making skills whether facing video sequences^27^ or in-situ (even if large effect size were observed in that case^28^).

As indicated earlier, Palao and colleagues^4^ claim that specialization with regard to the occupied position is of upmost importance in volleyball. Obviously, some generic skills like being able to jump or dive help all volleyball players to excel^1^. However, many specific skills are needed only for players in certain positions. For example, middle players typically jump higher, primarily having a role in blocking opposing attacks and a secondary role in attacks by their own team^29^. They constantly face the net and have to anticipate the opposing setter’s intention to maximize the chances that they will be on time for blocking. For their part, the hitters are versatile players specialized mostly in attacks, but also have strong abilities in both bumps and defense^4^. They have to get information on the quality of the set going in their direction and on the defenders’ and blockers’ position to maximize the chances of scoring. The liberos are players solicited only for bump and defense, never going to the net^1^. They mainly have to anticipate the direction of services and attacks from opposing teams. Finally, the setters are the designated *playmakers* and have clear decision-making responsibilities. Whenever their team performs a bump or a defense, they move under the ball and have to gather information firstly from the left (where the bump is coming from), and then quickly make a 180° head movement to the right to gather information on the opponent’s defense, keeping in mind at the same time where the ball and each of their hitters are. Then, they must quickly decide whom to pass the ball to in order to maximize the chances of scoring^4^.

The decisional responsibility inherent to the position of setter can even lead them to train differently from their teammates from a cognitive point of view^30^. For example, during official training with their teammates and coaches, they often have to practice making the most unpredictable decisions possible during drills that reproduce sequences of real games^30^. Outside of practice, they watch videos or discuss privately with their coaches to learn the strengths and weaknesses of the opposing teams’ defense to optimize their ball distribution^31^.

### The present study

The cognition-oriented training experienced by expert setters and the decisional responsibility they develop because of the cognitive demands of their position suggest that their context-specific perceptual-cognitive skills differ from that of other players. Knowing if these differences exist could later help coaches identify which visual cues or pattern are preferred by high-level players from various positions, which could in turn be used to detect and orient young players towards a position or another. Theoretically, comparing individuals with decision-making responsibilities with other experts from the same domain could deepen our understanding on the information processing skills in key decision-makers.

The goal of the present study is to compare key decision-makers with other experts from the same field. More specifically, the study verifies if expert setters, expert players from other positions and controls differ on their eye movements and anticipation skills facing various game situations: services, bumps, sets, attacks and blocks. Two hypotheses are formulated: (a) when facing all types of ball contact, setters will fixate on less elements, but these fixations will be on average longer than that of other players and controls and (b) setters, with each type of ball contact, will make more accurate predictions than other players and controls. No hypotheses are formulated regarding “when” and “where” the ocular fixations of each group will occur. This part of the study is exploratory. To answer these objectives, participants from the three groups will view volleyball video sequences that will stop right before ball contact. During the viewing, their eye movements on the screen will be recorded. Also, they will be invited to predict the direction of the ball after temporal occlusion. The main features of participants’ ocular fixations (number, duration, moment and location) as well as their anticipation efficiency to predict the direction of the ball correctly will be compared between groups.

## Results

### Number of fixations

**Figure 1:**
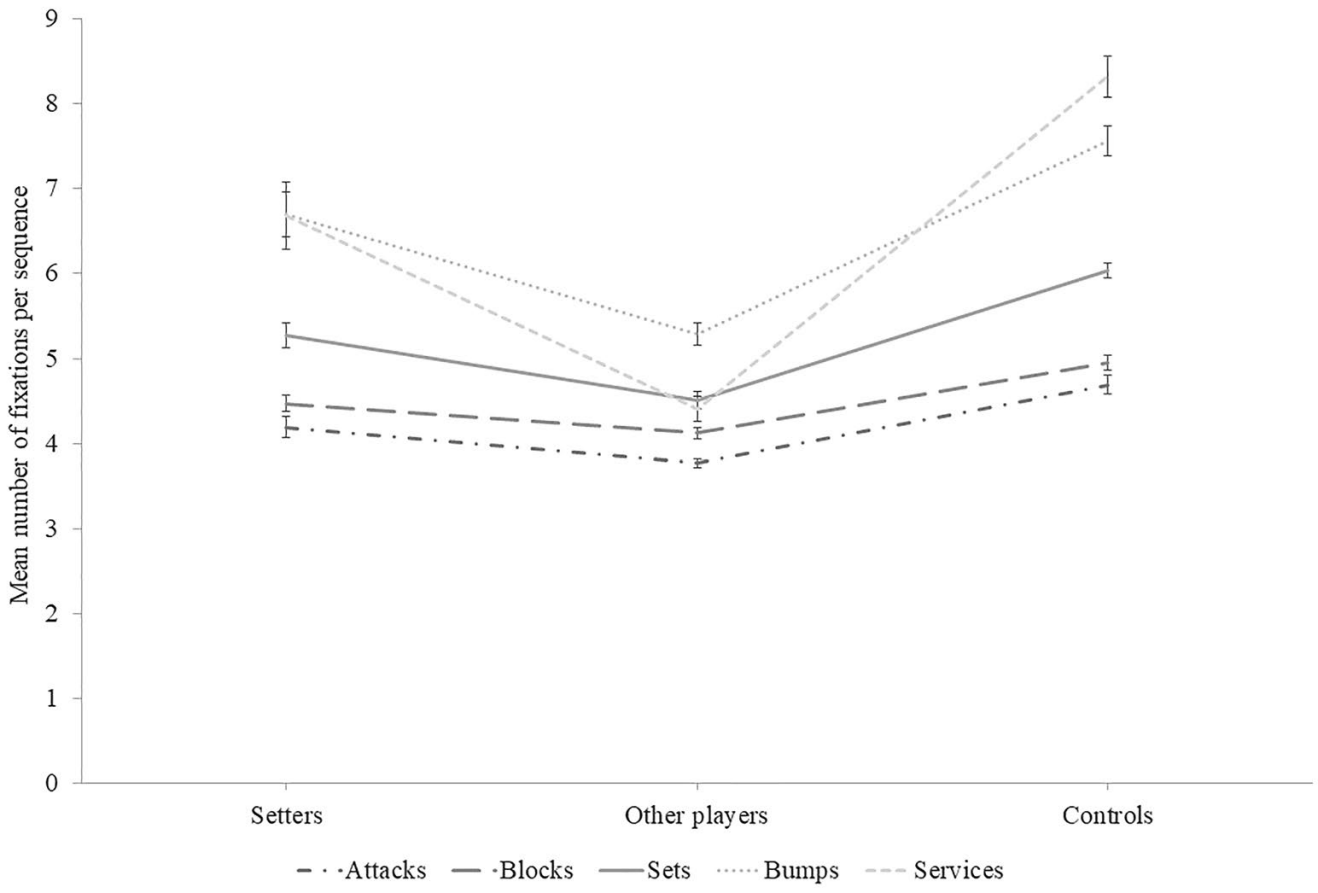
Mean number of fixations per sequence as a function of group and sequence types. Error bars represent the standard error of the means. Lines between data points are meant to facilitate the distinction between sequence types across groups, and not to presume of any quantitative link between groups.

Figure 1 illustrates the mean number of fixations per sequence as a function of groups and ball contact types. A Group × Ball contact type interaction effect was present for the mean number of fixations per sequence, χ^2^_w_(8) = 86.93, *p* < 0.001. Pairwise comparisons revealed that other players fixated less than controls for all types of ball contact (unilateral *p* < 0.001 for all five). Regarding services, bumps and sets, setters performed more fixations than other players (unilateral *p* < 0.01 for the three types). Setters fixated less than controls on services, sets and blocks (unilateral *p* = 0.026, *p* = 0.001, and *p* = 0.013 respectively), but these groups did not differ on bumps (unilateral *p* = 0.336). For attacks, setters did not differ from other players (*p* = 0.153), nor from controls (unilateral *p* = 0.113). For blocks, setters did not differ from other players (*p* = 0.338).

### Average fixation duration

**Figure 2:**
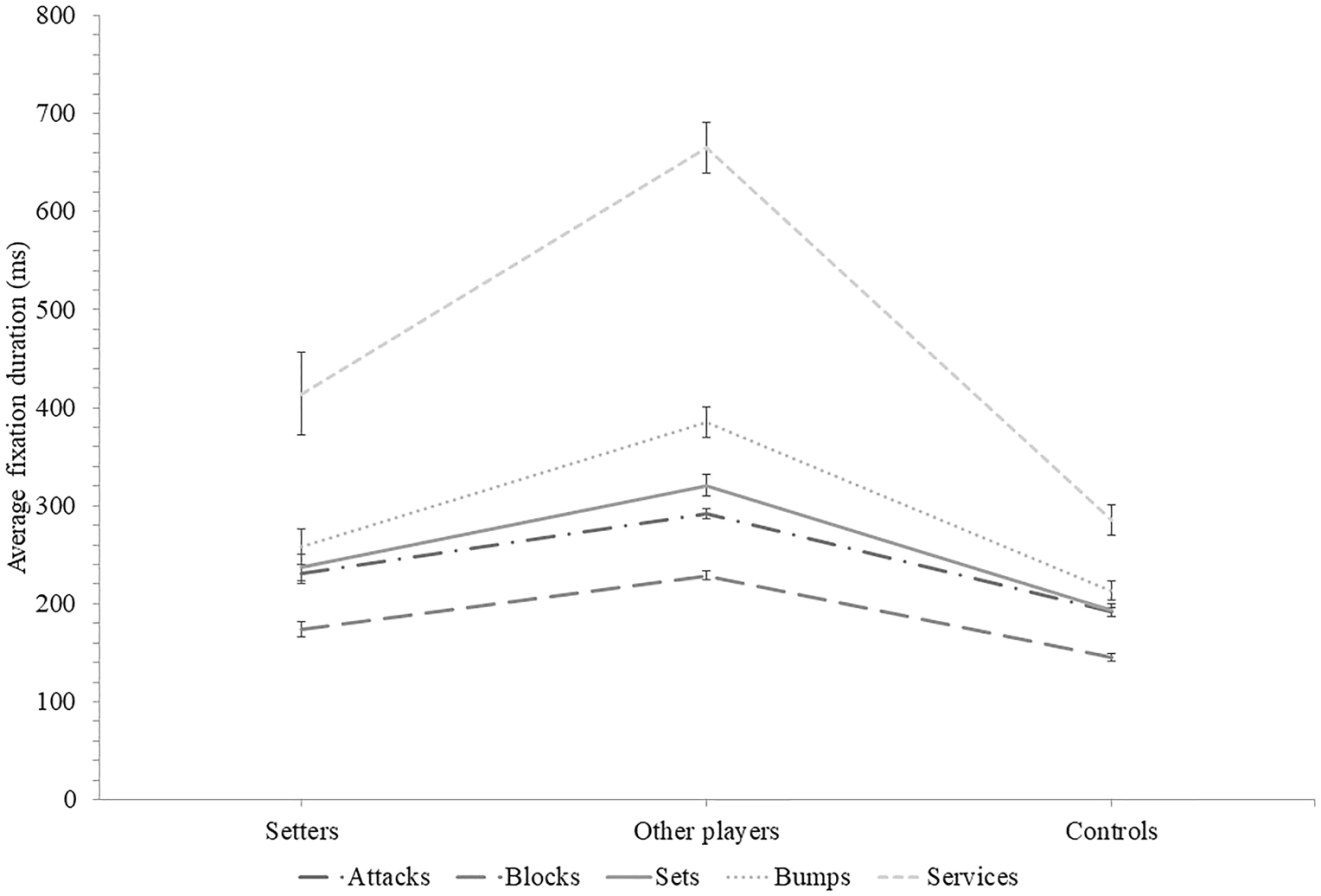
Average fixation duration as a function of groups and sequence types. Error bars represent the standard error of the means. Lines between data points are meant to facilitate the distinction between sequence types across groups, and not to presume of any quantitative link between groups.

Figure 2 illustrates the average fixation duration as a function of groups and sequence types. A Group × Ball contact type interaction effect was present for the average fixation duration, χ^2^_w_(8) = 48.84, *p* = 0.001. Pairwise comparisons revealed that other players’ average fixation durations were longer than that of setters and controls for all types of ball contact (for all comparisons, *p* < 0.01). Setters and controls only differed on attack sequences (unilateral *p* < 0.02; all other types of ball contact, unilateral *p* ≥ 0.06).

### Number of AOI fixated per sequence

**Figure 3:**
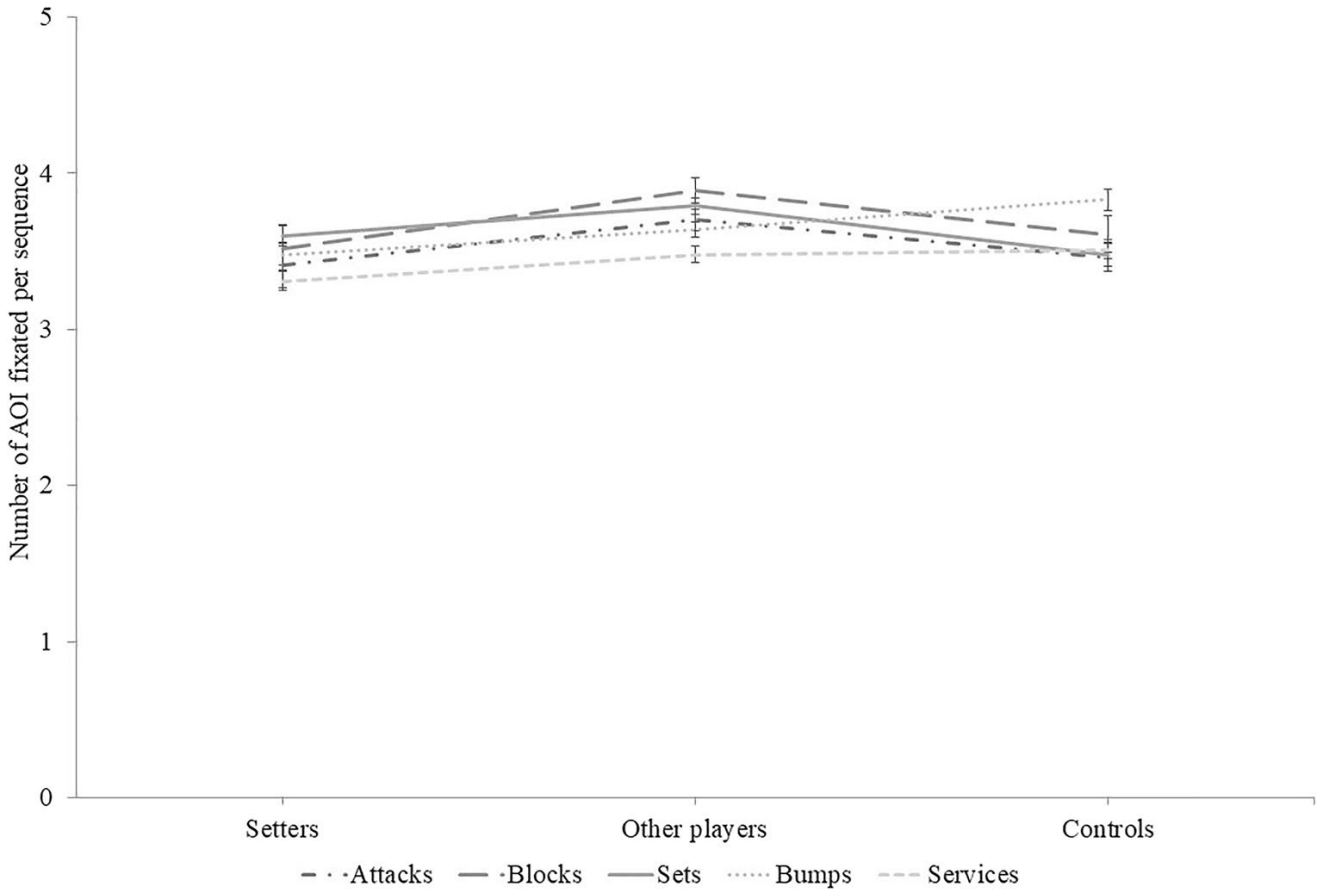
Mean number of AOIs fixated per sequence as a function of group and sequence types. Error bars represent the standard error of the mean. Lines between data points are meant to facilitate the distinction between sequence types across groups, and not to presume of any quantitative link between groups.

Figure 3 illustrates the mean number of AOI fixated per sequence as a function of groups and ball contact types.

#### Services

There was no effect of Group, χ^2^_w_(2) = 2.311, *p* = 0.315.

#### Bumps

There was no effect of Group, χ^2^_w_(2) = 5.494, *p* = 0.064.

#### Sets

There was no effect of Group, χ^2^_w_(2) = 5,424, *p* = 0.066.

#### Attacks

The effect of Group was statistically significant, χ^2^_w_(2) = 7,152, *p* = 0.028. However, pairwise comparisons revealed that no group differed significantly. Setters (*M* = 3.41; *S.E.* = 0.15) fixed as much AOIs as other players (*M* = 3.70; *S.E.* = 0.07; *p* = 0.198), and controls (*M* = 3.46; *S.E.* = 0.09; *p* = 1.000). Other players did not differ from controls as well (*p* = 0.086).

#### Blocks

The effect of Group was statistically significant, χ^2^_w_(2) = 7,003, *p* = 0.03. However, pairwise comparisons revealed that no group differed significantly. Setters (*M* = 3.52; *S.E.* = 0.14) fixed as much AOIs as other players (*M* = 3.89; *S.E.* = 0.08; *p* = 0.067), and controls (*M* = 3.61; *S.E.* = 0.12; *p* = 1.000). Other players did not differ from controls as well (*p* = 0.151).

### Number of correct predictions

**Figure 4:**
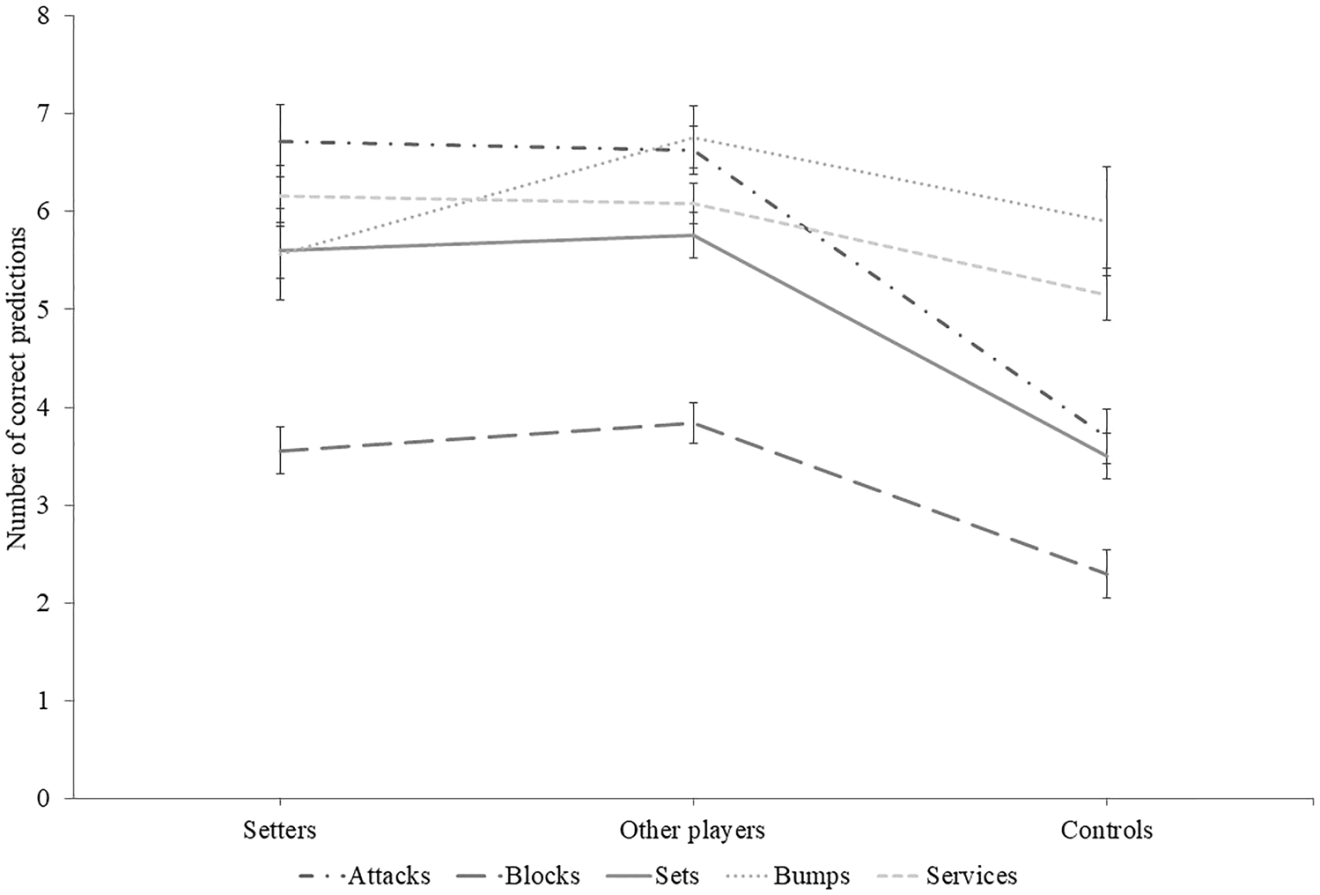
Mean correct predictions as a function of group and sequence types. Error bars represent the standard error of the mean. Lines between data points are meant to facilitate the distinction between sequence types across groups, and not to presume of any quantitative link between groups.

Figure 4 illustrates the mean number of correct predictions as a function of groups and sequence types. A Group × Ball contact type interaction effect was present for the number of correct predictions, χ^2^_w_(14) = 384.24, *p* < 0.001. Pairwise comparisons revealed that setters and other players did not differ for any type of ball contact (*p* = 1.000 for all types). However, both expert groups predicted correctly more often than controls for sets (*p* < 0.001 for both groups), attacks (*p* < 0.001 for both groups), and blocks (*p* = 0.022 and *p* < 0.001 respectively). No group differences were observed for bumps and services.

### Predicted probability that an AOI is fixated according to time

**Figure 5:**
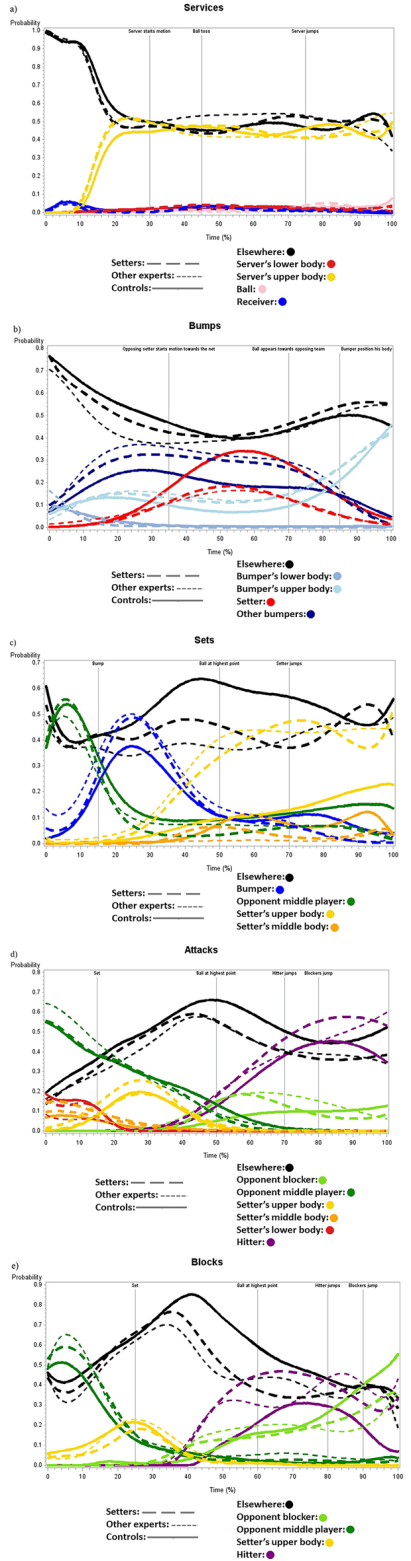
Predicted probability that an AOI is fixated as a function of time for each group and for and each type of sequence.

Figure 5a through Fig. 5e illustrate the predicted probability that an AOI is fixated according to moment of fixation and group for each type of ball contact. Table 1 presents the reasons (i.e., minimum QIC while keeping a full rank variance–covariance matrix) why each model was chosen to better fit the data. Note that results presented in this section are mostly descriptive as too many post-hoc analyses would have been required to test every observable difference at every time point, artificially inflating alpha error probability.

**Table 1:** Criteria for selecting the appropriate number of degrees for each model regarding predicted probability that an area of interest is fixated according to time and group for each type of sequence.

Degrees	Sequence type									
	Service		Bump		Set		Attack		Block	
	QIC	Full-rank	QIC	Full-rank	QIC	Full-rank	QIC	Full-rank	QIC	Full-rank
1	374,855.59	Yes	380,835.19	Yes	283,049.02	Yes	179,167.30	Yes	184,377.31	Yes
2	367,764.14	Yes	365,670.40	Yes	271,895.61	Yes	173,351.79	Yes	176,785.80	Yes
3	359,382.01	Yes	364,850.01	Yes	269,826.24	Yes	**173,108.91**	**Yes**	175,452.74	Yes
4	356,630.23	Yes	**364,414.04**	**Yes**	268,392.84	Yes	172,900.05	No	175,395.99	Yes
5	355,292.03	Yes	363,642.48	No	268,105.22	Yes			174,606.59	Yes
6	354,661.01	Yes			**267,500.16**	**Yes**			174,504.13	Yes
7	**354,384.35**	**Yes**			267,217.11	No			**174,319.08**	**Yes**
8	354,333.14	No							174,220.28	No

Models were chosen when QIC reached a minimum or when the matrix lost its full rank, whichever came first. Bold indicates the selected model. QIC = Quasi-likelihood under Independence Model Criterion.

#### Services

Figure 5a illustrates the analyses of services, where no AOI were removed. The analysis revealed a Group × Time interaction for the global test including all degree from linear to seven, χ^2^_w_(56) = 516.35, *p* < 0.001. All groups showed a similar pattern in gaze behaviors, as they first looked elsewhere with a probability greater than 0.95 until the server started his motion. Once the server began to move, this probability dropped to around 0.50. From this time point until occlusion, all groups either looked elsewhere (± 0.50) or at the server’s upper body (> 0.40).

#### Bumps

For bump sequences (Fig. 5b), only the ball AOI was removed from analysis since it represents 0.21% of the observation. A significant Group × Time interaction was found for the global test including all degree from linear to four, χ^2^_w_(32) = 136.45, *p* < 0.001. From onset and until the opposing setter was in motion, all groups had a decreasing probability of looking elsewhere, from above 0.70 to approximately 0.40. Both groups of experts started looking mainly at other receivers (probability greater than 0.30), whereas for controls, other receivers were also targeted, but less so (about 0.20 probability). Most notably, while both expert groups continued looking at other receivers when the opposing setter was moving, controls looked at the setter with a probability greater than 0.30. Finally, all groups looked at the receiver’s upper body before occlusion with a > 0.35 probability. No notable differences were observed between both expert groups.

#### Sets

For set sequences (Fig. 5c), two AOIs (i.e., ball [0.20%] and setter’s lower body [0.01%]) were removed. The global model (including every degree from linear to six) indicates a significant Group × Time interaction, χ^2^_w_(48) = 469.42, *p* < 0.001. At onset, there is a probability around 0.50 that the three groups fixate on the opponent middle player. Notable differences started to emerge at the moment of the bump, as both expert groups fixated the receiver at a ± 0.50 probability, whereas controls were at less than 0.40. From the moment the ball reached its highest point until the end of the sequence, both expert groups fixated at a ± 0.45 probability the setter’s upper body, whereas the controls did not show preference for a specific AOI (elsewhere: > 0.50; setter’s upper body: ± 0.20; opponent middle player: ± 0.10; setter’s middle body: < 0.10). No notable differences are observed between expert groups.

#### Attacks

One AOI (i.e., ball, 0.06%) was removed from the analysis regarding attacks (Fig. 5d). A significant Group × Time interaction was observed for the global model including every degree from linear to cubic, χ^2^_w_(36) = 129.17, *p* < 0.001. At onset, for the three groups, there is an approximate probability of 0.60 that the opponent middle player is fixated. After the set, all groups had a ± 0.20 probability to fixate the setter’s upper body. A strong (above 0.50) probability that fixations occurring between the set and the hitter’s jump are elsewhere was observed for all groups. From the moment the ball reached its highest point until occlusion, there is a growing probability for both expert groups to look either at the hitter (from below 0.30 to ± 0.55) or the opponent blocker (below 0.20). A similar pattern was observed for controls, except that after the blocker jumped, they started to abandon the hitter, and rather looked elsewhere (± 0.50). No notable differences are observed between expert groups.

#### Blocks

Figure 5e illustrates the analysis of blocks. The ball, the setter’s lower body and middle body were the three AOIs removed from this analysis since they represented only 0.19%, 0.76% and 0.80% of the dataset respectively. A significant Group × Time interaction was observed for the global model including every degree from linear to seven, χ^2^_w_(56) = 1521.51, *p* < 0.001. At onset, there is a ± 0.60 probability for both expert groups and a ± 0.50 probability for controls to look at the opponent middle player. At the moment of the set, a similar pattern across all groups was observed, as they either looked at the setter’s upper body (± 0.20 probability) or elsewhere (above 0.60). Once the set was directed to the hitter, differences across all groups started to emerge. Setters forsake in part “elsewhere” in favor of either the hitter (probability increasing up to above 0.40 and gradually decreasing to below 0.30) or the opponent blocker (probability gradually increasing from ± 0.10 to above 0.30). For their part, other players also partially forsake “elsewhere” and looked at the opponent blocker (probability gradually increasing from ± 0.10 to above 0.30), but their probability (above 0.40) to look at the hitter peaked much later than setters (i.e., at the blocker’s jump rather than at the hitter’s jump). The most notable difference concerns controls, as they almost completely forsake the hitter once he jumped in favor of the opponent blocker (probability increasing from below 0.30 to above 0.50).

## Discussion

The aim of the study was to compare the context-specific perceptual-cognitive skills of experts with key decision-making responsibilities with that of other experts from the same field. More specifically, expert setters in volleyball were compared with expert players from other positions and controls on eye movements and anticipation efficacy when facing video sequences of all types of ball contact found in a typical game (i.e., services, bumps, sets, attacks and blocks). It was hypothesized that setters would perform fewer but longer ocular fixations and would anticipate more efficiently the follow-up actions than other players and controls when facing all types. These hypotheses took root on the setters’ cognition-oriented role and training^30^. They were also ingrained on previous literature showing that, compared to novices, expert athletes tend to perform fewer but longer ocular fixations, when facing short non-life-size video sequences taken from their area of expertise^6^, while anticipating more accurately what is coming next^14^.

### Number of fixations and duration

Partly contrary to expectations, setters did fixate less than controls on three out of five types of ball contact (i.e., services, sets and blocks). However, they performed more fixations than other players on services, bumps and sets. In addition, setters’ average fixation duration was shorter than other players on all types, and did not differ from controls except on attacks. These results can be surprising at first sight because they could illustrate that setters, even if they cumulated more than 4000 h of volleyball training (similar to other players), their information processing while facing context-specific situations resembles that of controls. But does it really? The question is relevant because results from the present study also indicate that setters and other players did not differ in terms of anticipation skills on any type of ball contact, but they were both better than controls on sets, attacks and blocks (which partly confirm the second hypothesis stating that setters would be better than other players and controls, and that other players would be better than controls). Therefore, the interpretation that setters and controls resemble with regards to their perceptual-cognitive skills while facing volleyball situations appears unlikely. Even more so if we consider the well-known expert advantage regarding perceptual-cognitive skills facing context-specific situation in sports^6^, and specifically in volleyball in a more nuanced fashion^19–21^.

It is more probable that results from the present study support the idea that setters constitute a subgroup of experts, having their own way of processing information. Indeed, during a typical game, setters have to gather information firstly from the bump trajectory, and then quickly gather information from the opponent’s defense, all the while knowing where the ball and each of their hitters are. Players from other positions do not require these frequent eye and head movements with great amplitude: they usually gather information in front of them, with a much smaller span of field of view. For example, middle players are facing the net, trying to anticipate the intention of the opposing setter and hitter, all information being available within a slight left–right rotation of the head. Therefore, it is plausible that setters gather as much information from shorter but more frequent fixations than other players, while not losing anticipation efficacy. Another explanation to the current results could lie in the absence of consensus on the definition of the duration of an ocular fixation itself. Many authors agree that the fixation location can correspond to the information being processed^8^, and once an efficient target has been locked in, it is preferable to stay on it to extract as many information as possible^6,12,13^. However, it can also be described as a moment where information extraction is difficult^32^. Because setters fixated for shorter periods in the present study, it is possible that they not only have facility identifying relevant targets, but also extracting information from them. Therefore, they can save time and allocate it to gather information on other targets. These results lend support to the idea that different roles and responsibilities among experts from a same field (in volleyball and maybe in general) can serve as an important factor to identify key decision-makers. Specifically in terms of sports expertise, this is the main novelty of the present study.

### Exploring the AOIs fixated

In order to try to identify where the differences between setters and controls lie and explore alternative explanations to the current results, analyses of the number of AOIs fixated were conducted. The results illustrate that setters did not differ from controls on all types of ball contact. Therefore, the number of AOIs fixated does not appear a promising way to distinguish setters from controls.

Pursuing the same objective of distinguishing setters and controls, analyses of the moment of fixation on each AOI were conducted. Results suggest that setters sometimes differ from controls. This avenue of research seems more exploitable. Indeed, even if an expertise effect can be found in other players with discrete and more typical eye movements measures (i.e., number and fixation duration), those measures failed at finding an expertise effect between setters and controls. However, a dynamic analysis of eye movements led to some extent to such an expertise effect (e.g., during bump sequences, controls payed more attention than both expert groups to the opposing setter once he started moving, even if the setter is not a player involved in bumps). These results suggest that within experts, attentional patterns can differ with respect to playing positions, as various measures are required to observe a difference with controls.

Another possibility is that the main difference between setters and controls rather lies within the location of fixations, and especially those occurring “elsewhere”. A subsequent analysis on X and Y coordinates of “elsewhere” fixations as a function of groups and time (not shown in “Results” section; see supplementary material) was conducted to test this hypothesis. Results revealed a group effect (or time by group interaction effect) on each sequence type. When looking closer at the location of the “elsewhere” fixations according to group and time, it appears that for service sequences, setters may be quicker at fixating relevant AOIs or visual pivot between AOIs (i.e., server’s upper body and the space between the ball and the server) than other experts and controls. Indeed, their number of “elsewhere” fixations located completely out of the action appears lower once the server starts his motion. A similar interpretation can be expressed regarding set sequences, but for both expert groups taken together as opposed to controls. Indeed, even if the three groups seem to use a visual pivot throughout those sequences, the experts’ visual pivot is placed between relevant AOIs quicker than controls. For example, when the ball is at its highest point following the bump, experts’ visual pivot is between the setter and the opponent middle player, whereas controls still placed their pivot between the receiver and the opponent middle player, even if the receiver was not involved in the action anymore. No group differences regarding the use of a visual pivot or fixating on a specific AOI seem to emerge from bump, attack and block sequences. These results suggest that experts (and maybe more specifically setters when considering service sequences) may be faster at identifying relevant targets and, when needed, at fixating on functional spaces between them to gather information from their peripheral vision^10^. Even if the eye-tracking device cannot measure peripheral vision (i.e., information processing away from the fixation point), it has been shown that athletes sometimes rely on peripheral vision by anchoring their gaze between AOIs^9^. Setters, when deciding to which hitter to send the ball to, have to gather information relative to opponent players’ positioning more than they need to capture precise biomechanical information. This positioning can easily be gathered from a well-trained peripheral vision. This interpretation is plausible, but should be taken cautiously, as it is mostly based on an attentional pattern found on service sequences, whereas even set sequences do not seem to distinguish setters from other players in their use of a visual pivot.

Of note is that the results from the present study regarding set video sequences replicate to some extent those of Piras and colleagues^19^, where the setter’s upper body appeared to be a relevant visual cue to anticipate his intention. Results from the present study regarding the “when” and the “where” of the other four types of ball contact can be seen as novelty and replication is needed before informing athletes and coaches on preferable attentional pattern. However, it seems that the server’s upper body is the most important attentional cue to look at in order to predict the direction of a service, and this seems to hold true quickly at the beginning of service sequences, all the way through occlusion. Concerning attacks and blocks, looking at the opponent hitter appears relevant to facilitate anticipation, especially when occlusion is near. It could have been informative to separate AOIs with respect to the hitter’s body in order to know which part is the most relevant. Note that anecdotal examination of the fixations in the present study suggests that hitters’ upper body seems relevant. Finally, regarding bumps, looking at the receiver’s upper body right before the ball contact seems to be an adequate attentional target.

Opponents’ upper body has been demonstrated as a key visual cue in other domains like martial arts^33–35^. In this field, it is seen as a way to anchor the gaze between multiple relevant body parts of the opponent (e.g., both hands of the opponent) in order to gather information from many sources at the same time. In volleyball, it is unlikely that this is the reason why athletes fixate opponent’s upper body. Indeed, the fast and frequent transient changes in the game dynamic where more than one opponent (plus the ball^20,23^) has to receive attention give very little time to anchor the gaze in the middle of the torso of one opponent. This is especially true in the present study where the absolute size of the stimuli (i.e., all of the opponents’ bodies) on the screen were small, leaving little space to anchor the gaze between the hands of the opponent. Because of the nature of volley-ball, it is more probable that the orientation of the opponent’s shoulders is what athletes’ are looking for. According to the present study, this seems to hold true in all game situations. Shoulders orientation gives information on the direction of the future hit. Unless there is a last-minute twist of the wrist (which could lead to an error), the direction of the ball in serves, bumps, sets and attacks will most often follow the direction of the shoulders. It can hold true in blocks as well, but to a lesser extent, as the direction of the ball after a block is mostly random. Overall, the exploratory results of the present study in relation with dynamic eye movements point out that in volleyball, whichever type of ball contact is occurring, looking at the involved player’s upper body (i.e., probably shoulders’ orientation) right before the action appears informative.

### Limitations, strengths and future studies

The present study has limitations preventing generalization of results. Since setters have to gather information from their left and their right while being perpendicular to the net, including video sequences from the left of the court pointing toward the right could have been informative in order to differentiate setters from other players. Also, the present study took place in the laboratory, limiting potential generalization of the results to actual game because in-situ studies in sports sometimes yield different results^6^. The measure of anticipation efficacy in the present study also suffers limitations. One is the depth problem, caused by the fact that only three possible outcomes reduce the refinement of the measures (especially if we compare to the nine that were initially planned). Another limitation is related to the number of trials; accuracy was measured in steps of 10% accuracy for each type of ball contact. Finally, the high number of “elsewhere” fixations suggests that another operational definition of the AOIs might have been useful. The supplementary material accompanying this manuscript intends to cover part of this limitation.

The present study also has methodological strengths. Of note is the sample size. Most studies in sports (and more specifically in volleyball) compare experts and novices with groups comprised with 10–15 participants at most. The sample recruited for the present study increases statistical power, and thus the generalization of results. In addition, the laboratory setting, where distractions and stimuli were controlled, encourages isolation of perceptual-cognitive processes.

Several future research projects can be fueled by the present study. First, researchers should try to replicate the present results in-situ or by presenting stimuli from the left of the court pointing right. This would allow verifying if and how more ecological situation modulate eye movements and anticipation efficacy of setters and other players. Second, the results from the present study invite researchers to continue investigating perceptual-cognitive differences between experts based on their position. Indeed, since setters resemble controls on some variables, but resemble other players on some others, it appears relevant to dig deeper in this phenomenon in order to identify what characterizes best this subgroup of experts. Also, research could be conducted on the anticipation of the position of other players, rather than on the ball, as it is more in line with the tasks performed by setters on the field. Finally, researchers could verify if perceptual-cognitive differences exist in other domain based on decision-making responsibilities (e.g., quarterback in American football versus other players^36^).

## Conclusion

Setters and other players look mostly at the same thing at the same time and anticipate volleyball actions more efficiently than controls. However, setters tend to fixate more and for shorter periods than other players, a pattern that is close to the one found in controls. These results could be due to the setters’ propensity to move the head suddenly during actual games or to their capacity to extract as much information as other players and controls, but in shorter amount of time. Interestingly, when appropriate, experts use a visual pivot to gather information from their peripheral vision, but this strategy is not systematic, and most importantly, not always specific to experts, as no observable differences in visual pivot were found between groups for bump, attack and block sequences. Overall, the results support, with nuance, the idea that setters constitute a subgroup of expert volleyball players regarding perceptual-cognitive skills, but provide no reason to believe that their anticipation capabilities differ from that of other players. These results open the way to the study of decision-making responsibilities as an important factor to identify if and how key decision-makers in sports (and other domains) may differ from their equally experienced peers.

## Methods

### Participants

The study sample (*N* = 82) is composed of three groups: expert setters (*n* = 26), expert volleyball players from other positions (*n* = 36; other players), and physically active university students (*n* = 20; controls). Table 2 describes the three groups in terms of age, sex distribution, number of years/hours of experience in volleyball and the number of other practiced sports in their lifetime. To be included in the study, participants from both expert groups had to (a) play volleyball in a university or college division 1 or 2 team, and (b) have played or trained at least 4000 h of volleyball, while participating in at least eight other sports activities (organized or not) in their life^37^. Note that the latter criterion has been relaxed as the recruitment went on since some participants accumulated about 3000 h of volleyball while having participated in 12 other activities or conversely, accumulated about 8000 h, without taking part in at least eight other activities. It seemed that excluding such experienced volleyball players would result in important data loss. As for controls, they had to have accumulated less than 1000 h of volleyball in their life, while self-identify as active individuals (not necessarily being elite in any sport). All participants had to be 18 years of age or older, have a normal or corrected-to-normal vision using contact lenses, report no history of neurological/psychiatric disorder, and take no medication such as antidepressants, anxiolytics or neuroleptics.

**Table 2:** Sociodemographic characteristics of setters, other players and controls.

Variables	Groups		
	Setters (*n* = 26)	Other players (*n* = 36)	Controls (*n* = 20)
% Women	38.46^a^	47.22^a^	50.00^a^
Mean age (SD)	19.46^a^ (1.39)	19.75^a^ (2.00)	23.85^b^ (2.35)
Mean number of years playing volleyball (SD)	7.65^a^ (1.96)	7.58^a^ (2.64)	0.00^b^ (0.00)
Mean number of hours of volleyball in lifetime (SD)	4098.58^a^ (1407.89)	4648.00^a^ (2044.99)	141.50^b^ (153.06)
Mean number of other sports practiced in lifetime (SD)	9.08^a^ (1.55)	8.81^a^ (1.31)	10.90^a^ (3.04)

Different letters in superscript indicate significant difference after Bonferonni correction (*p* < .05) as compared to other groups, whereas same letter indicate no difference.

#### Recruitment

Two recruitment methods were used to complete both expert groups. First, sports directors from colleges and a university in the [Name of the city] region were contacted by telephone to make an appointment and present the study. A written agreement was concluded with them to obtain the contact information of their volleyball head coaches. Coaches were contacted in order to obtain permission to present the study during a typical practice session. During this practice, athletes were informed of the study and its implications (i.e., one experimental session of about 45 min), and their voluntary participation was solicited. A sheet was provided to each athlete where they could indicate their interest and contact information. Athletes agreeing to participate were contacted by telephone to determine their eligibility. Second, the first author solicited the participation of expert volleyball players during a nationwide tournament. Eligibility of players interested was verified on the spot, and those eligible took part in the experimental session immediately. As for controls, a recruiting email was sent to students at [Name of University] using automatic mailing lists. Students agreeing to participate answered the email by giving their telephone number. They were called to verify their eligibility. The ethics committee of the Université Laval in research with humans approved this study (approbation number: 2017-001 A-1 R-1/05-09-2018) and all research was performed in accordance with relevant guidelines. No participants under the age of 18 were involved.

### Material

Whether participants came to the laboratory or participated during the tournament, the experimental session took place in a soundproof room free from distractions, on an Intel Core i-7 computer running the Windows 8 system with a 22-in. computer screen. The eye movements of the participants on the screen were tracked at 120 Hz using a Tobii X3-120 device (0.4° accuracy and 0.24° precision; similar devices are used in sport psychology research^38^). The *Tobii Pro Lab* software version 1.27 (Tobii Pro, Stockholm, SWE; https://www.tobiipro.com/product-listing/tobii-pro-lab/) was used to program the experiment and present the video sequences to the participants.

#### Video sequences

Two types of video sequences were filmed, using a Nikon 30 Hz camera, and presented to the participants. All sequences were edited using the *Shotcut* software version 17.01 (Meltytech, LLC.; https://shotcut.org/). First, soccer penalty shots from the goalkeeper’s perspective were filmed, and served as training for the general functioning of the experiment. To design the video sequences, a camera operator was hired. A soccer field, a soccer goal and a soccer ball were used. A senior AAA soccer player with 21 years of experience as a player and five years as a coach shot the penalty shots 11 m away from the camera, which was placed on the center of the goal line. Out of 54 penalty shots filmed, the player chose the ten most representative ones.

Second, for the experimental phase, the video sequences illustrated volleyball sequences from the point of view of a back-line player in the center of the field. The camera was elevated two meters above the back line of the court. A standard volleyball court (18 m × 9 m), a volleyball net (height 2.43 m) and a standard volleyball ball (65 to 67 cm in circumference, 294 to 318 millibars of air pressure) were used. A camera operator was also hired to shoot the footages. Eleven former male college and university players (retired for a maximum of two years) were invited to shoot the video sequences. Three hundred and ninety-seven ball contacts were judged usable by the first author (having 11 years of experience as a volleyball player and five years as a coach). This number included 47 services, 73 bumps, 125 sets, 112 attacks and 40 blocks. The discrepancy between the numbers of usable ball contacts can be explained by the fact that more than one set and attack can be filmed from a same rally. In addition, blocks are typically rarer than other ball contacts.

The first author then kept the 20 ball contacts of each type (total of 100) judged most representative according to (a) the clarity of the technical gesture, and (b) the equivalent distribution of the ball between the sequences (e.g., seven sets to the left, six sets in the center and seven sets to the right were selected at this stage). These 100 sequences were shown to two certified coaches with over 30 years of experience each in volleyball. They had to evaluate the representativeness of the sequences on a Likert type scale ranging from 0 (*not representative*) to 7 (*perfectly representative*). The 10 sequences of each type (total of 50) with the highest average scores were kept for the experimentation (for a similar selection method, see^39,40^). Before the experiment, sequences were randomized, but all participants viewed the sequences in the same order.

### Measures

#### Eligibility questionnaire

This homemade questionnaire consists of 11 questions (four with short answers and seven with dichotomous responses) that determine the eligibility to participate in the study (see criteria described above). For questions requiring further thoughts (e.g., number of hours of volleyball during lifetime), the interviewer verbally helped the athletes with the calculation.

#### Sociodemographic questionnaire

This self-administered homemade questionnaire consists of seven questions (one dichotomous, three short-answer, and four multi-responses) that collect general information such as sex, age, marital status, education, main occupation, height (meters) and weight (kilograms).

#### Eye movements

The eye movements on the computer screen were recorded during the presentation of all the experimental video sequences. Each type of video sequence was divided into AOIs (see Table 3). Fixations occurring outside the identified AOIs corresponded to “elsewhere”. A region was considered fixed if the angular velocity of the eyes relative to the stimuli was 30°/s or less for at least 100 ms^39,41^. Number of fixations, fixation time, AOI fixated and the moment of fixation of those AOIs were measured. Note that the moment of fixation was transformed into percentage of time passed since the beginning of the sequence (i.e., the sequences were separated in 100 time frames). This is because video sequences were not exactly all the same length. For example, the shortest bump sequence was 1231 ms, whereas the longest was 2957 ms (span of 1726 ms, which represents the longest span across all five sequence types). Importantly, main events during each sequence occurred roughly at the same percentage of viewing time, limiting the potential impact of this data manipulation on the results and their interpretation.

**Table 3:** Areas of interest (AOIs) for each type of video sequences.

AOIs	Sequence type				
	Service	Bump	Set	Attack	Block
Hitter				X	X
Ball	X	X	X	X	X
Opponent blocker				X	X
Opponent middle player			X	X	X
Setter		X			
Setter (lower body)			X	X	X
Setter (middle body)			X	X	X
Setter (upper body)			X	X	X
Receiver	X		X		
Receiver (upper body)		X			
Receiver (lower body)		X			
Other receivers		X			
Server (upper body)	X				
Server (lower body)	X				

In some cases, two or three AOIs were fixated at the same moment because they overlapped (e.g., blocker from one side of the net and hitter from the other side, as the camera was placed in the back-court). In these cases, only one AOI was retained for analysis. To determine the retained AOI, the AOIs fixated the moment before and after were verified. If one of the overlapping AOIs was the same as those fixated right before or after, it was the one retained. In rare cases, the AOI fixated before and after differed from the overlapping AOIs. In these cases, to determine the AOI retained for analysis, video sequences were manually examined by superimposing the participant’s gaze behavior on them. Then, a decision was made (based on first author’s volleyball experience) on the most likely visual cue that was attended to at that moment between the two overlapping AOIs. For example, towards the end of an attack sequence, if both the hitter’s and the blocker’s AOIs were activated, the hitter was kept for analysis as he is the main player involved.

#### Anticipation efficacy

The accuracy of the prediction made using the numeric pad of the keyboard when occluding each video sequence was measured. For each video sequence, participants had to indicate the region of the screen they thought the ball would go after the occlusion. For example, pressing “7” on the numeric pad would indicate that the participant thought the ball would go on the top left corner of the screen. Note that this measure was recoded as “Left” (answers 1, 4 and 7), “Center” (answers 2, 5 and 8) and “Right” (answers 3, 6 and 9) because many participants reported having trouble transposing the numeric pad (flat on the table) to the computer screen (vertical), creating a depth problem with the measure.

### Procedure

Eligible and interested participants came to the [Name of laboratory and University] (or in an isolated room if they were recruited during the tournament). Upon arrival, their written informed consent was obtained and they completed the sociodemographic questionnaire. They then sat about 60 cm away from the computer screen (i.e., the screen described visual angles of approximately 45° in width and 26° in height). The first author explained that they would watch 60 video sequences (10 soccer, then 50 volleyball, all preceded by a slide announcing what type of ball contact is coming) that stop 120 ms^39^ before contact of the player with the ball. From this temporal occlusion, they had to indicate where they thought the ball would go using the numeric keypad. They were given an example on exactly what to do, where they should press “9” on the keypad if they thought the ball would go in the top right corner of the screen. After training, the first author asked participants to keep to a minimum their head movements, and a nine-point calibration of the eye-tracking device was performed. This calibration invited the participant to follow, with their eyes, a moving point on the screen, which made nine changes of direction. Participants received a 10 [Name of currency] monetary compensation to cover travel expenses.

### Research design and data analysis

This study corresponds to an *ex post facto* quasi-experimental design. Analyses were conducted on the *IBM SPSS 24* software (IBM Corp., Armonk, NY; https://www.ibm.com/analytics/spss-statistics-software). The data analysis for this paper requiring further coding (i.e., “when” and “where” the fixations were) was generated using SAS/STAT software, Version 9.4 of the SAS System for Windows. Copyright 2020 SAS Institute Inc. SAS and all other SAS Institute Inc. product or service names are registered trademarks or trademarks of SAS Institute Inc., Cary, NC, USA.

Percentages, means and standard deviations are used to describe the sample. One-way ANOVAs (with Bonferonni post-hoc tests) and independence Pearson’s Chi-square tests were run on the descriptive statistics to verify group homogeneity (Table 1). Generalized Estimated Equations (GEE) analyses were conducted on all dependent variables with Bonferonni post-hoc comparisons with respect to the group as a between-subject factor (expert setters, other players and controls). GEE were chosen to take into account dependency between the observations of each subject and to allow flexibility in the choice of the variance–covariance matrix as well as the distribution of the data. The dependent variables correspond to eye movements (number of fixations per sequence, average duration of each fixation per sequence, number of AOIs fixated per sequence, AOI fixed; the high number of “elsewhere” fixations presented in the Results section (Fig. 5) was caused by AOIs being drawn too small at the beginning of the analysis process. Larger AOIs could not be redrawn because approximately half the dataset suffered an irreversible technical problem) and number of correct predictions made (out of 10) at temporal occlusion. Because groups differed in terms of age, correlation analyses were run between participants’ age and all the dependent variables. No correlation was found; therefore, age was not included as a covariate in any analysis.

For *number of fixations*, *number of AOIs fixated*, and *number of correct predictions*, a Poisson distribution, a logarithmic link function, and an exchangeable matrix were selected. For the *average fixation duration*, a normal distribution, a logarithmic link function, and an exchangeable matrix were selected. For these models, the type of ball contact (services, bumps, sets, attacks, blocks) was added as a within-subject factor, except for *number of AOIs fixated* where models were run separately with respect to the type of video sequence because the AOIs were not the same from one type of sequence to another (Table 2). For *AOI fixated*, a multinomial distribution, a generalized logit link function and an independent matrix were selected. A few AOIs were excluded because their frequencies of fixation were smaller than 1% of the total number of fixations. In these models, the moment of fixation before temporal occlusion (ranging from 0 to 100%) is added as a within subject factor, as well as its interaction with the group. The number of degrees for the polynomial effect of moment of fixation was chosen in order to minimize QIC. However, in some cases, the QIC needed to be higher than the minimum to preserve a full rank variance–covariance matrix. For all analyses, the alpha threshold is fixed at 0.05.

## Supplementary information

Supplementary Information.
