# Supplementary material for: Decision-making and dynamics of eye movements in volleyball experts
Source: Sci Rep. 2020 Oct 14;10:17288. doi: 10.1038/s41598-020-74487-x (PMC7560879; doi:10.1038/s41598-020-74487-x)
Supplement: Supplementary file 1 — Supplementary Information. [file 41598_2020_74487_MOESM1_ESM.docx]

Decision-making and dynamics of eye movements in volleyball experts

**Authors**

*Daniel Fortin-Guichard, M.A.^1^

Tel: 418-656-2131, ext. 404624

E-mail: daniel.fortin-guichard.1@ulaval.ca

Vincent Laflamme, B.A.^1^

School of Psychology, Université Laval

Tel: 418-656-2131, ext. 404624

E-mail: vincentlaflamme.vl@gmail.com

Anne-Sophie Julien, M.Sc.^2^

Tel: 418-656-2458

E-mail: anne-sophie.julien@mat.ulaval.ca

Christiane Trottier, Ph.D.^3^

Tel: 418 656-2131, ext. 404203

E-mail: christiane.trottier@fse.ulaval.ca

Simon Grondin, Ph.D.^1^

Tel: 418-656-2131, ext. 406227

E-mail: simon.grondin@psy.ulaval.ca

^1^ School of Psychology, Université Laval, Québec, Canada

^2^ Department of Mathematics and Statistics, Université Laval, Québec, Canada

^3^ Department of Physical Education, Université Laval, Québec, Canada

**Supplementary material**

The analyses presented as supplementary material are Generalized estimated equations of the X and Y coordinates of the “elsewhere” ocular fixations according to time between the start of the video sequence and the temporal occlusion. Analyses were conducted separately for every type of ball contact. All main effects of the group and time are shown. When main effect of group was detected, simple effect tests are also reported. However, only the significant interactions are reported. If Group by Time interaction is detected, the moment in the video sequence where there are differences are presented. Figures illustrate the temporal evolution of the coordinates. For X coordinates, a higher value indicates that more fixations are directed towards the right of the screen. For Y coordinates, a higher a value indicates that more fixations are directed towards the top of the screen.

After each figure, a table is provided presenting, for each group, whether the “elsewhere” fixations were (1) attributed to an AOI (and which one), (2) between AOIs (i.e., potentially used as a visual pivot between multiple AOIs), (3) in the vicinity of the main action or (4) completely outside the action. Each table focuses on moments where there were evident changes in either the X or Y coordinates for a given type of sequence. For example, the figure related to the X coordinates of the Bumps sequences depicts an inversion around 30% of viewing time. Therefore, the moment between 25% and 35% of viewing is reported in the table. Since it was not possible to redraw the AOIs (because of a technical problem described in the Methods section of the article), we used the minimum and maximum X and Y coordinates of the fixations observed within each AOI at every targeted moment of every type of sequence to redraw the AOIs artificially. Then, the distances (in pixels in both X and Y directions) between each “elsewhere” fixation and the newly drawn AOIs were calculated. Note that because the targeted moments comprised 10% of viewing time and the AOIs were moving, some “elsewhere” fixations now fall within an AOI.

The criteria to determine the location of each “elsewhere” fixation were hierarchical. First, when an “elsewhere” fixation was within one newly drawn AOI, it was attributed to this AOI. In some instances where only one or two AOIs had been fixated during a targeted moment, “elsewhere” fixations were considered as inside the AOI if it was no further away than 10% of the size of the AOI (in pixels). Second, if an “elsewhere” fixation was not inside the new AOIs, but at a distance smaller than 300 pixels from the two nearest AOIs, the “elsewhere” was considered between the two AOIs. This threshold was chosen based on the distribution of the distances, where there was a clear drop in the frequency of “elsewhere” fixations further away than 300 pixels. Third, if the two nearest AOIs from an “elsewhere” fixation came from the same player on screen (e.g., setter’s upper body and setter’s middle body) this fixation could not be considered between them. Therefore, in those instances, these AOIs were fused and the distance with the third nearest AOI was verified with a threshold of 400 pixels, as the distribution of the third nearest AOIs showed a drop around this value. When no third AOI had been fixated, the “elsewhere” fixations were considered in the vicinity of the main action. Finally, the “elsewhere” fixations were considered outside the action when they were not within 300 pixels of the nearest AOI.

**Services**

**X coordinates of the “elsewhere” fixations**

| Significant effects | df | χ² | *p* |
| --- | --- | --- | --- |
| Group | 2 | 0.79 | .675 |
| Time | 1 | 19.11 | <.001 |
| Time^2 | 1 | 7.14 | .008 |

**
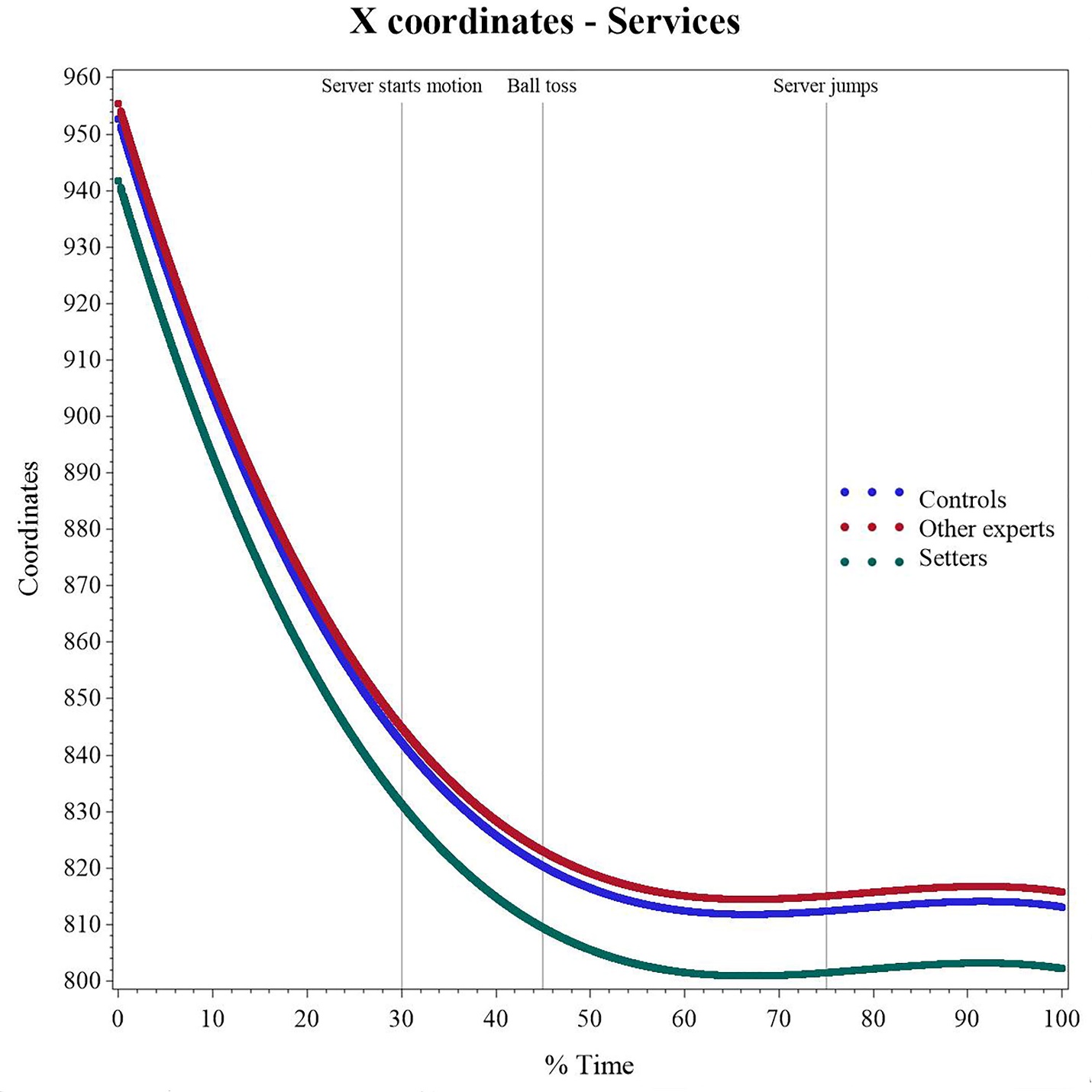
**

**Y coordinates of the “elsewhere” fixations**

| Significant effect | df | χ² | *p* |
| --- | --- | --- | --- |
| Group | 2 | 1.47 | .479 |
| Time | 1 | 56.86 | <.001 |
| Time*Group | 2 | 5.99 | .050* |
| Time^2 | 1 | 46.53 | <.001 |
| Time^2*Group | 2 | 7.13 | .028* |
| Time^3 | 1 | 36.81 | <.001 |
| Time^4 | 1 | 28.21 | <.001 |

*No difference between groups at 10% of elapsed time. At 30% of elapsed time (i.e., when servers start their motion), only a difference between controls and other experts was found, z = 2.35, *p = .*019. At 50% of elapsed time (i.e., ball toss), only a difference between controls and other experts was found, z = 2.42, *p = .*015. No differences were found from 70% of elapsed time onward.

**
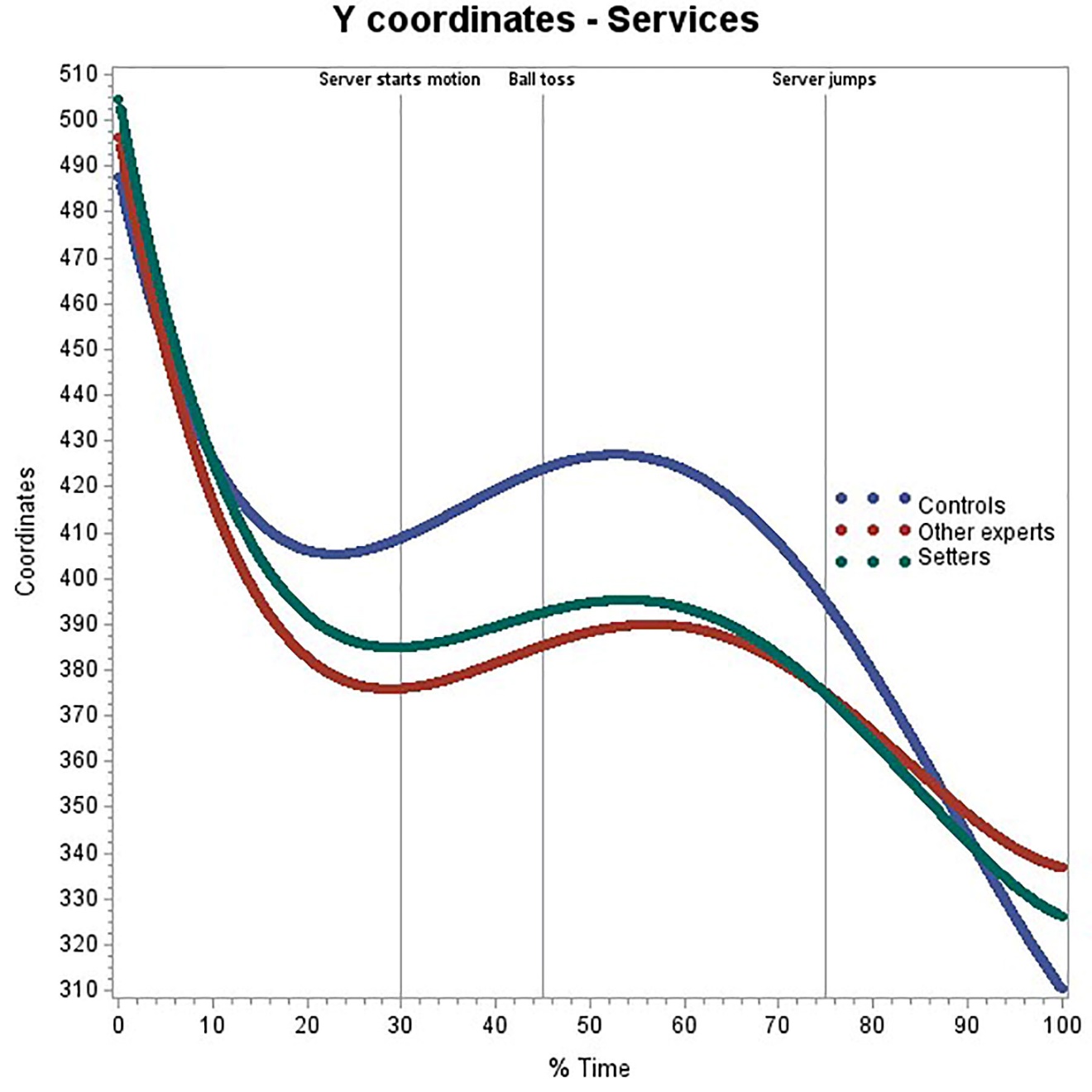
**

**Distribution of the location of the “elsewhere” fixations during the service sequences**

|  | Moments (in % of viewing time) | | | | | | | | | | | | | | |
| --- | --- | --- | --- | --- | --- | --- | --- | --- | --- | --- | --- | --- | --- | --- | --- |
|  | 5 | | |  | 25 | | |  | 55 | | |  | 95 | | |
| Location | S | O-E | C |  | S | O-E | C |  | S | O-E | C |  | S | O-E | C |
| Attributed to an AOI |  |  |  |  |  |  |  |  |  |  |  |  |  |  |  |
| Receiver | 24.0 | 17.9 | 20.2 |  | 0.0 | 0.0 | 0.8 |  | 0.0 | 1.0 | 0.9 |  | 0.6 | 0.5 | 0.0 |
| Server’s upper body | 0.8 | 2.8 | 2.0 |  | 52.9 | 47.3 | 42.3 |  | 38.9 | 39.0 | 36.4 |  | 54.4 | 58.0 | 56.7 |
| Server’s lower body | 0.8 | 0.0 | 0.0 |  | 0.0 | 0.0 | 0.0 |  | 1.5 | 1.5 | 1.7 |  | 1.8 | 3.5 | 0.7 |
| Ball | 0.0 | 0.0 | 0.0 |  | 0.0 | 0.5 | 4.9 |  | 2.3 | 1.5 | 1.7 |  | 5.3 | 5.5 | 6.4 |
| Total | 25.6 | 20.7 | 22.2 |  | 52.9 | 47.8 | 48.0 |  | 42.7 | 43.0 | 40.7 |  | 62.1 | 67.5 | 63.8 |
|  |  |  |  |  |  |  |  |  |  |  |  |  |  |  |  |
| Between two AOIs |  |  |  |  |  |  |  |  |  |  |  |  |  |  |  |
| Receiver – Server’s upper body | 19.2 | 22.9 | 15.2 |  | 0.7 | 1.1 | 1.6 |  | 3.1 | 2.0 | 1.7 |  | 0.0 | 0.5 | 0.0 |
| Receiver – Server’s lower body | 13.6 | 13.4 | 15.2 |  | 0.0 | 0.0 | 0.0 |  | 0.0 | 0.0 | 0.0 |  | 0.0 | 0.0 | 0.7 |
| Receiver – Server’s whole body | 4.0 | 5.0 | 3.0 |  | 13.0 | 9.8 | 16.3 |  | 10.7 | 5.0 | 3.4 |  | 0.0 | 0.0 | 0.0 |
| Server’s upper body – Ball | 0.0 | 0.0 | 0.0 |  | 16.7 | 15.2 | 14.6 |  | 26.7 | 25.0 | 25.4 |  | 29.0 | 23.0 | 24.1 |
| Server’s lower body – Ball | 0.0 | 0.0 | 0.0 |  | 0.0 | 0.0 | 0.0 |  | 0.0 | 0.5 | 0.0 |  | 0.0 | 0.0 | 0.0 |
| Server’s whole body - Ball | 0.0 | 0.0 | 0.0 |  | 0.0 | 2.2 | 1.6 |  | 1.5 | 2.5 | 2.5 |  | 4.7 | 5.0 | 7.8 |
| Receiver – Ball | 0.0 | 0.0 | 0.0 |  | 0.7 | 0.0 | 0.0 |  | 0.0 | 0.0 | 0.0 |  | 0.0 | 0.0 | 0.0 |
| Total | 36.8 | 41.3 | 33.4 |  | 31.1 | 28.3 | 34.1 |  | 42.0 | 35.0 | 33.0 |  | 33.7 | 28.5 | 32.6 |
|  |  |  |  |  |  |  |  |  |  |  |  |  |  |  |  |
| In the vicinity of the main action | 0.0 | 0.0 | 0.0 |  | 7.3 | 7.1 | 4.9 |  | 7.6 | 8.0 | 9.3 |  | 3.0 | 3.5 | 3.6 |
|  |  |  |  |  |  |  |  |  |  |  |  |  |  |  |  |
| Out of the action | 37.6 | 38.0 | 44.4 |  | 8.7 | 16.9 | 13.0 |  | 7.6 | 14.0 | 17.0 |  | 1.2 | 0.5 | 0.0 |

*Notes.* S = Setters. O-E = Other experts. C = Controls. AOI = Area of interest. The lines regarding the server’s whole body presents instances where the
AOIs of parts of the server’s body had to be fused and the third nearest AOI had to be verified. Each column totalizes 100% of the “elsewhere” fixations
of each group at a given moment, but due to rounding to one decimal it may not always add up to 100%. The lines presenting the totals only refer to their
respective subsection and should not be considered in the calculation of the columns totals.

**Bumps**

**X coordinates of the “elsewhere” fixations**

| Significant effects | df | χ² | *p* |
| --- | --- | --- | --- |
| Group | 2 | 1.81 | .404 |
| Time | 1 | 29.74 | <.001 |
| Time^2 | 1 | 15.01 | <.001 |
| Time^3 | 1 | 8.05 | .005 |
| Time^4 | 1 | 4.55 | .033 |

**
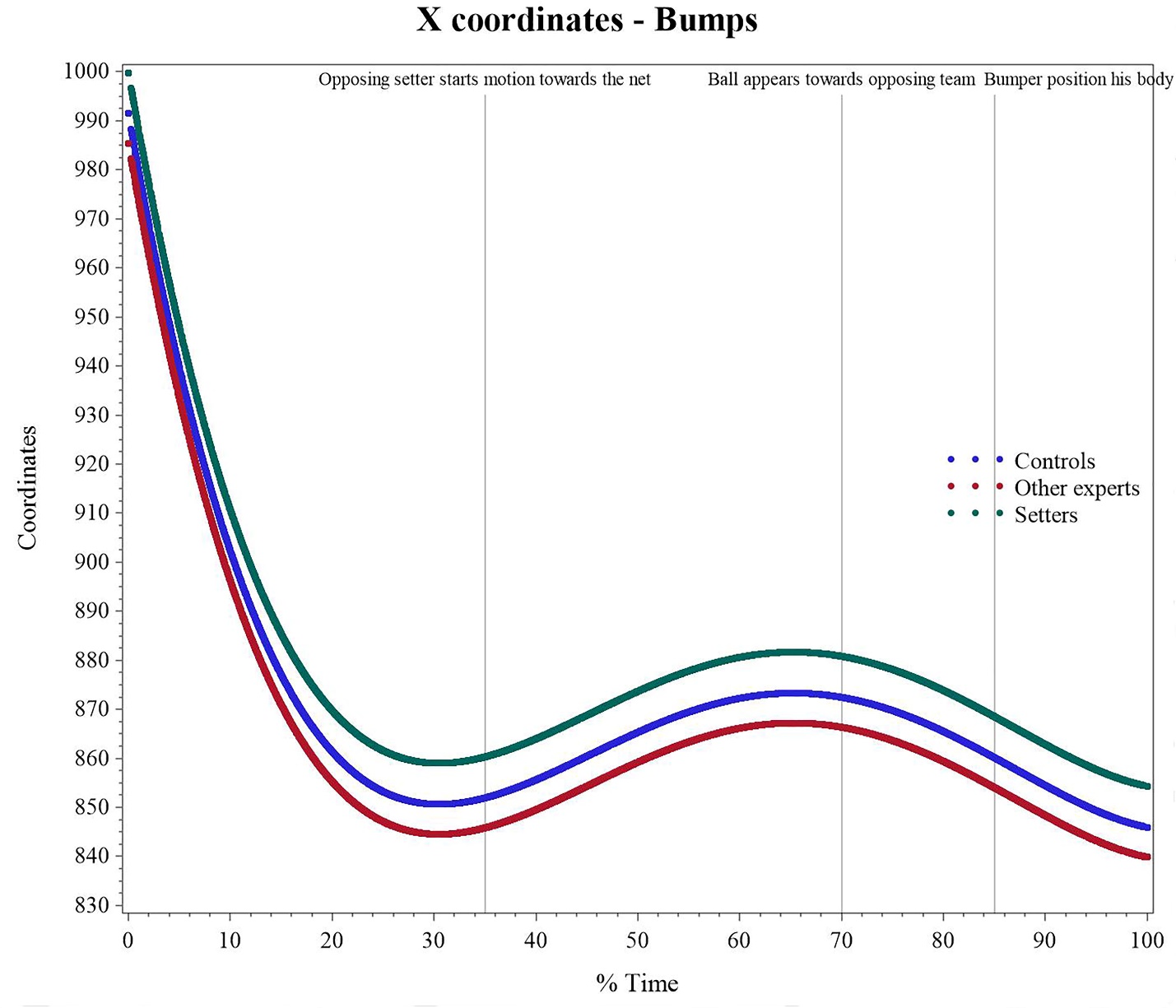
**

**Y coordinates of the “elsewhere” fixations**

| Significant effect | df | χ² | *p* |
| --- | --- | --- | --- |
| Group | 2 | 3.63 | .163 |
| Time | 1 | 24.45 | <.001 |
| Time*Group | 2 | 16.79 | .002* |
| Time^2 | 1 | 23.48 | <.001 |
| Time^2*Group | 2 | 16.13 | .003* |
| Time^3 | 1 | 20.76 | <.001 |
| Time^4 | 1 | 21.80 | <.001 |

*No difference between the groups at 10% of elapsed time. At 30% of elapsed time (i.e., when opposing setters start motion towards the net), only the difference between setters and controls was significant, z = 2.49, *p =.*013. At 50% of elapsed time (i.e., ball in the direction of opposing court, not yet visible), differences were found between (1) setters and controls, z = 3.67, *p* < .001 and (2) controls and other experts, z = 2.06, *p = .*040. At 70% of elapsed time (i.e., balls appear on screen), only a difference between setters and controls was found, z = 3.32, *p* < *.*001.

**
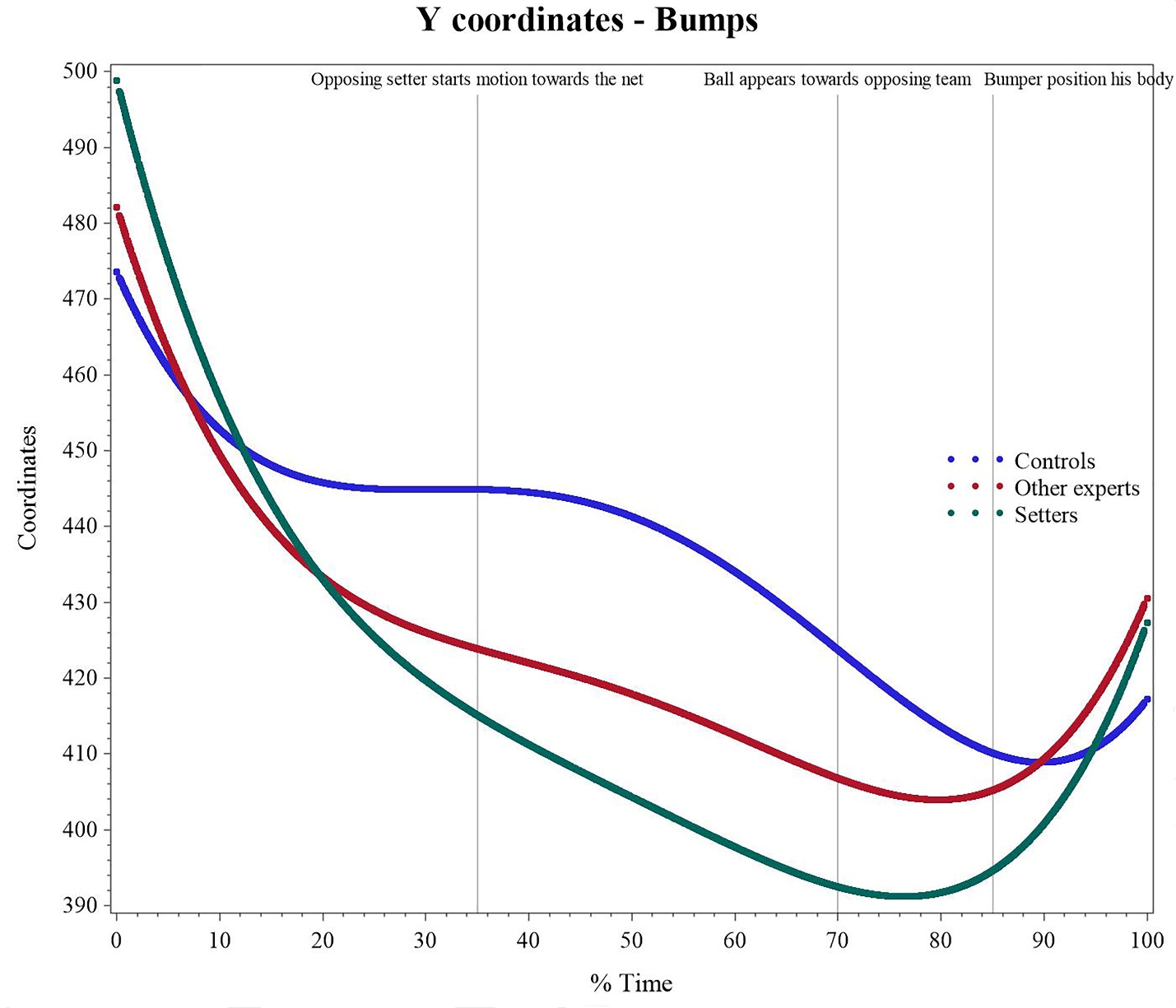
**

|  | Moments (in % of viewing time) | | | | | | | | | | | | | | | | | | |
| --- | --- | --- | --- | --- | --- | --- | --- | --- | --- | --- | --- | --- | --- | --- | --- | --- | --- | --- | --- |
|  | 5 | | |  | 30 | | |  | 50 | | |  | 65 | | |  | 95 | | |
| Location | S | O-E | C |  | S | O-E | C |  | S | O-E | C |  | S | O-E | C |  | S | O-E | C |
| Attributed to an AOI |  |  |  |  |  |  |  |  |  |  |  |  |  |  |  |  |  |  |  |
| Other receivers | 21.4 | 29.6 | 25.3 |  | 38.1 | 33.3 | 37.6 |  | 46.1 | 39.4 | 48.6 |  | 36.2 | 36.1 | 40.0 |  | 0.6 | 4.1 | 4.4 |
| R’s upper body | 38.2 | 33.0 | 32.7 |  | 26.1 | 38.1 | 18.4 |  | 17.2 | 22.5 | 15.9 |  | 40.9 | 34.2 | 32.7 |  | 69.2 | 64.7 | 55.2 |
| R’s lower body | 10.4 | 10.2 | 10.7 |  | 2.2 | 0.7 | 1.6 |  | 0.0 | 0.0 | 0.0 |  | 0.0 | 0.0 | 0.0 |  | 0.0 | 0.0 | 0.0 |
| Ball | 0.0 | 0.0 | 0.0 |  | 0.0 | 0.0 | 0.0 |  | 0.0 | 0.0 | 0.0 |  | 0.0 | 0.0 | 0.0 |  | 12.2 | 11.9 | 9.6 |
| Total | 70.0 | 72.8 | 68.7 |  | 66.4 | 72.1 | 57.6 |  | 63.3 | 61.9 | 64.5 |  | 77.1 | 70.3 | 72.7 |  | 82.0 | 80.7 | 69.2 |
|  |  |  |  |  |  |  |  |  |  |  |  |  |  |  |  |  |  |  |  |
| Between two AOIs |  |  |  |  |  |  |  |  |  |  |  |  |  |  |  |  |  |  |  |
| R’s upper body – Other receivers | 2.3 | 4.9 | 4.0 |  | 6.7 | 6.1 | 5.6 |  | 10.9 | 13.4 | 3.7 |  | 8.7 | 14.6 | 11.8 |  | 2.9 | 4.6 | 8.8 |
| R’s lower body – Other receivers | 0.6 | 0.0 | 0.7 |  | 0.0 | 0.0 | 0.0 |  | 0.0 | 0.0 | 2.8 |  | 0.0 | 0.0 | 0.0 |  | 0.0 | 0.0 | 0.0 |
| R’s whole body – Other receivers | 5.8 | 6.3 | 3.3 |  | 1.5 | 1.3 | 4.0 |  | 3.9 | 2.8 | 3.7 |  | 0.0 | 0.6 | 0.0 |  | 0.0 | 0.0 | 0.0 |
| R’s upper body – Ball | 0.0 | 0.0 | 0.0 |  | 0.0 | 0.0 | 0.0 |  | 0.0 | 0.0 | 0.0 |  | 0.0 | 0.0 | 0.0 |  | 12.2 | 13.3 | 15.4 |
| Ball – Other receivers | 0.0 | 0.0 | 0.0 |  | 0.0 | 0.0 | 0.0 |  | 0.0 | 0.0 | 0.0 |  | 0.0 | 0.0 | 0.0 |  | 1.2 | 0.5 | 1.5 |
| Total | 8.7 | 11.2 | 8.0 |  | 8.2 | 7.4 | 9.6 |  | 14.8 | 16.2 | 10.2 |  | 8.7 | 15.2 | 11.8 |  | 16.3 | 18.4 | 25.7 |
|  |  |  |  |  |  |  |  |  |  |  |  |  |  |  |  |  |  |  |  |
| In the vicinity of the main action | 2.9 | 2.4 | 2.7 |  | 1.5 | 2.7 | 0.8 |  | 0.0 | 0.7 | 0.0 |  | 0.0 | 0.0 | 0.0 |  | 0.0 | 0.0 | 0.0 |
|  |  |  |  |  |  |  |  |  |  |  |  |  |  |  |  |  |  |  |  |
| Out of the action | 18.5 | 13.6 | 20.7 |  | 23.9 | 17.7 | 32.0 |  | 21.9 | 21.1 | 25.2 |  | 14.2 | 14.6 | 15.5 |  | 1.7 | 0.9 | 5.2 |

**Distribution of the location of the “elsewhere” fixations during the bump sequences**

*Notes.* S = Setters. O-E = Other experts. C = Controls. AOI = Area of interest. R. = Receiver. The line regarding the receiver’s whole body presents instances where the AOIs of parts of the receiver’s body had to be fused and the third nearest AOI had to be verified. Each column totalizes 100% of the “elsewhere” fixations of each group at a given moment, but due to rounding to one decimal it may not always add up to 100%. The lines presenting the totals only refer to their respective subsection and should not be considered in the calculation of the columns totals.

**Sets**

**X coordinates of the “elsewhere” fixations**

| Significant effects | df | χ² | *p* |
| --- | --- | --- | --- |
| Group | 2 | 12.79 | .002* |
| Time | 1 | 17.22 | <.001 |
| Time^2 | 1 | 36.69 | <.001 |
| Time^3 | 1 | 40.04 | <.001 |
| Time^4 | 1 | 39.65 | <.001 |

*Differences between (1) setters and controls: z = 4.21, *p* < .001, and (2) other experts and controls: z = 3.46, *p* < .001. No difference between setters and other experts: z = 0.75, *p* = .451.

**
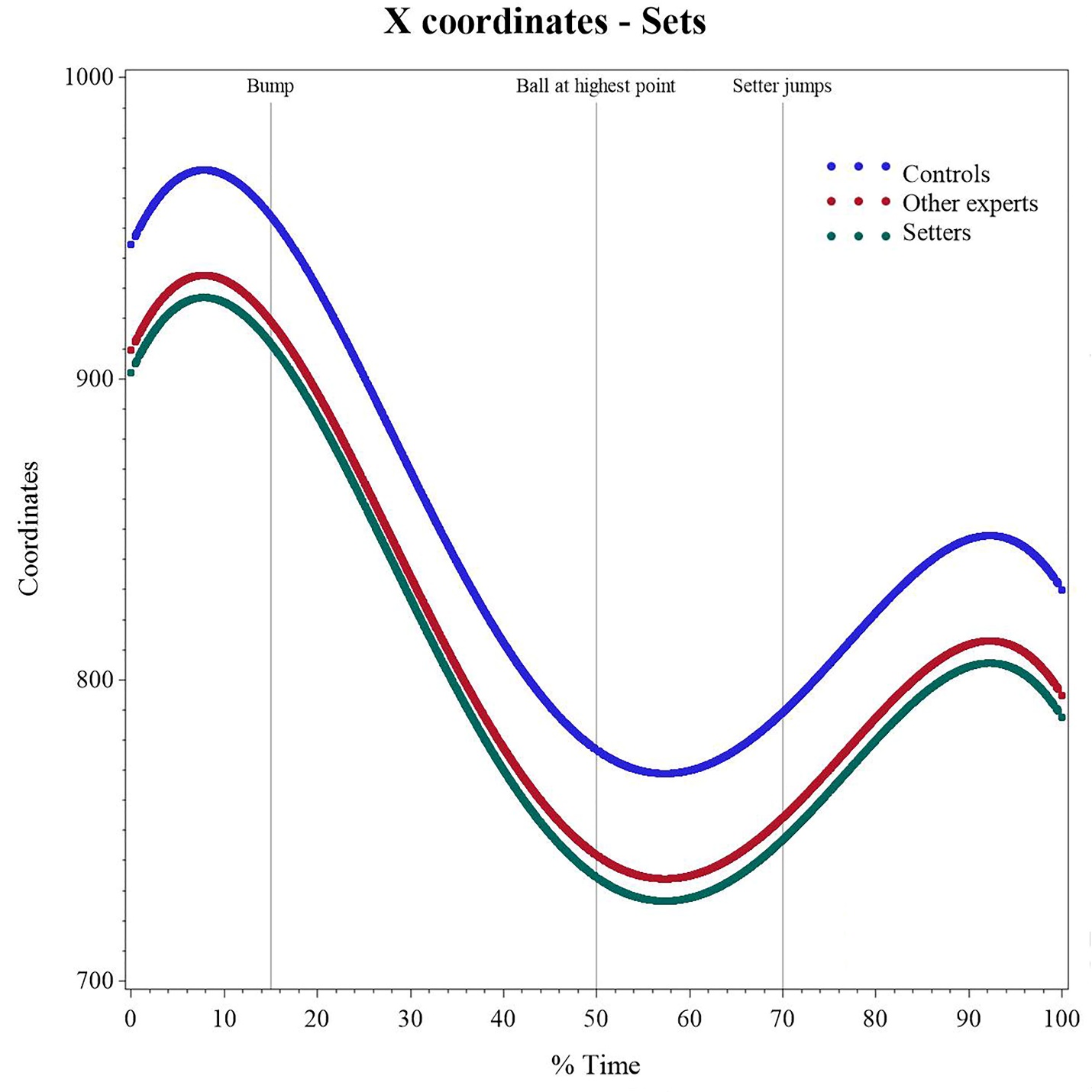
**

**Y coordinates of the “elsewhere” fixations**

| Significant effect | df | χ² | *p* |
| --- | --- | --- | --- |
| Group | 2 | 4.70 | .095 |
| Time | 1 | 60.69 | <.001 |
| Time^2 | 1 | 60.25 | <.001 |
| Time^3 | 1 | 61.52 | <.001 |

**
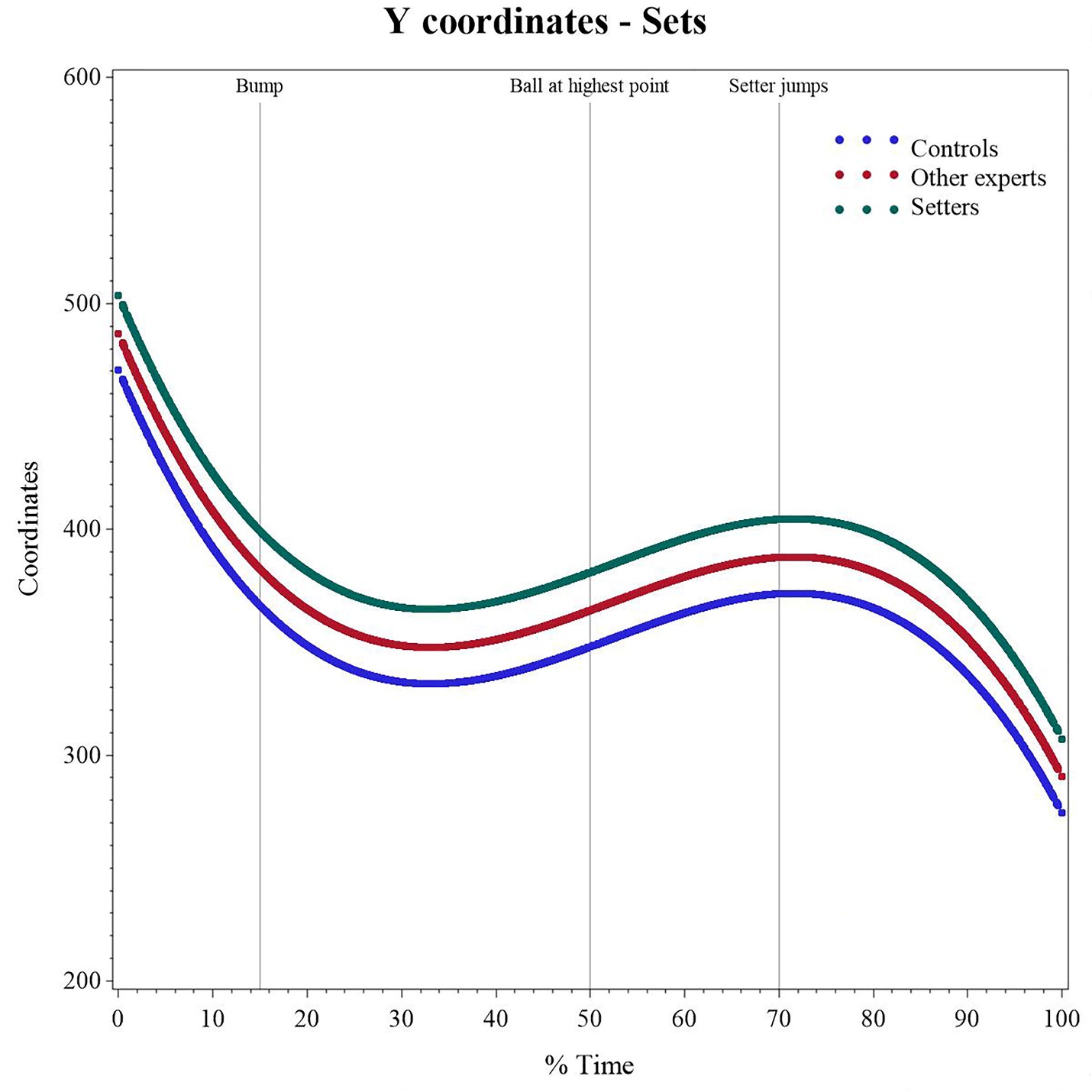
**

|  | Moments (in % of viewing time) | | | | | | | | | | | | | | | | | | |
| --- | --- | --- | --- | --- | --- | --- | --- | --- | --- | --- | --- | --- | --- | --- | --- | --- | --- | --- | --- |
|  | 10 | | |  | 30 | | |  | 55 | | |  | 75 | | |  | 95 | | |
| Location | S | O-E | C |  | S | O-E | C |  | S | O-E | C |  | S | O-E | C |  | S | O-E | C |
| Attributed to an AOI |  |  |  |  |  |  |  |  |  |  |  |  |  |  |  |  |  |  |  |
| S’s upper body | 0.0 | 0.7 | 0.0 |  | 11.8 | 9.0 | 8.3 |  | 32.1 | 17.7 | 17.5 |  | 46.2 | 32.0 | 25.6 |  | 43.1 | 36.1 | 18.0 |
| S’s middle body | 0.0 | 0.0 | 0.0 |  | 0.0 | 0.6 | 0.0 |  | 1.5 | 0.7 | 1.6 |  | 1.7 | 0.0 | 2.4 |  | 1.2 | 1.4 | 5.4 |
| O middle player | 12.6 | 17.2 | 9.5 |  | 5.5 | 4.2 | 4.1 |  | 3.1 | 4.3 | 4.0 |  | 4.2 | 6.6 | 4.8 |  | 2.4 | 0.9 | 1.8 |
| Receiver | 14.6 | 4.8 | 14.3 |  | 22.8 | 27.0 | 21.5 |  | 3.1 | 4.3 | 4.0 |  | 1.7 | 3.3 | 2.4 |  | 0.0 | 0.9 | 1.8 |
| Ball | 1.9 | 2.1 | 4.8 |  | 0.0 | 0.0 | 0.0 |  | 0.0 | 0.0 | 1.6 |  | 0.0 | 0.0 | 1.6 |  | 0.0 | 0.5 | 0.0 |
| Total | 29.1 | 24.8 | 28.6 |  | 40.1 | 40.8 | 33.9 |  | 39.8 | 27.0 | 28.7 |  | 53.8 | 41.9 | 36.8 |  | 46.7 | 39.8 | 27.0 |
|  |  |  |  |  |  |  |  |  |  |  |  |  |  |  |  |  |  |  |  |
| Between two AOIs |  |  |  |  |  |  |  |  |  |  |  |  |  |  |  |  |  |  |  |
| S’s upper body – Receiver | 18.5 | 21.4 | 22.6 |  | 23.6 | 25.8 | 22.3 |  | 10.7 | 17.0 | 9.5 |  | 11.8 | 14.9 | 8.0 |  | 14.4 | 13.3 | 7.2 |
| S’s upper body – Ball | 1.0 | 2.1 | 0.0 |  | 0.0 | 0.0 | 0.0 |  | 0.0 | 0.0 | 0.0 |  | 0.0 | 0.0 | 0.0 |  | 6.0 | 11.0 | 6.3 |
| S’s whole body – Ball | 0.0 | 0.0 | 0.0 |  | 0.0 | 0.0 | 0.0 |  | 0.0 | 0.0 | 0.0 |  | 0.0 | 0.0 | 0.0 |  | 0.6 | 0.9 | 0.9 |
| O middle player – Ball | 3.9 | 4.8 | 3.6 |  | 0.0 | 0.0 | 0.0 |  | 0.8 | 0.0 | 1.6 |  | 0.0 | 0.0 | 0.0 |  | 0.0 | 0.0 | 0.0 |
| Receiver – S’s whole body | 5.8 | 2.8 | 2.4 |  | 0.0 | 1.2 | 0.0 |  | 13.0 | 8.5 | 7.9 |  | 1.7 | .2 | 2.4 |  | 0.0 | 3.2 | 0.0 |
| Receiver – Ball | 14.6 | 13.1 | 16.7 |  | 0.0 | 0.0 | 0.8 |  | 0.8 | 0.0 | 1.6 |  | 0.0 | 0.0 | 0.0 |  | 0.0 | 0.0 | 0.0 |
| O middle player – S’ upper body | 0.0 | 0.0 | 0.0 |  | 4.7 | 3.6 | 0.0 |  | 3.1 | 7.1 | 6.4 |  | 18.5 | 24.9 | 21.6 |  | 19.2 | 20.1 | 27.0 |
| O middle player – S’ middle body | 1.9 | 7.6 | 1.2 |  | 0.0 | 0.0 | 0.0 |  | 0.0 | 0.7 | 0.8 |  | 0.0 | 0.0 | 0.0 |  | 0.0 | 0.0 | 0.0 |
| O middle player – S’ whole body | 0.0 | 0.0 | 0.0 |  | 4.7 | 1.8 | 2.5 |  | 16.8 | 14.2 | 6.4 |  | 3.4 | 2.8 | 4.8 |  | 7.8 | 6.9 | 9.9 |
| O middle player – Receiver | 24.3 | 22.8 | 23.8 |  | 24.4 | 22.8 | 36.4 |  | 8.4 | 6.4 | 24.6 |  | 4.2 | 8.3 | 20.0 |  | 1.8 | 2.3 | 16.2 |
| Total | 70.0 | 74.6 | 70.3 |  | 57.4 | 55.2 | 62.0 |  | 53.6 | 53.9 | 58.8 |  | 39.6 | 51.1 | 56.8 |  | 49.8 | 57.7 | 67.5 |
|  |  |  |  |  |  |  |  |  |  |  |  |  |  |  |  |  |  |  |  |
| In the vicinity of the main action | 0.0 | 0.0 | 0.0 |  | 0.0 | 0.6 | 0.0 |  | 5.3 | 9.2 | 4.8 |  | 2.5 | 2.2 | 3.2 |  | 1.8 | 2.7 | 5.4 |
|  |  |  |  |  |  |  |  |  |  |  |  |  |  |  |  |  |  |  |  |
| Out of the action | 1.0 | 0.7 | 1.2 |  | 2.4 | 3.6 | 4.1 |  | 1.5 | 9.9 | 7.9 |  | 4.2 | 2.8 | 3.2 |  | 1.8 | 0.0 | 0.0 |

**Distribution of the location of the “elsewhere” fixations during the set sequences**

*Notes.* S = Setters. O-E = Other experts. C = Controls. O = Opponent. AOI = Area of interest. The lines regarding the setter’s whole body presents instances where the AOIs of parts of the setter’s body had to be fused and the third (or fourth) nearest AOI had to be verified. Each column totalizes 100% of the “elsewhere” fixations of each group at a given moment, but due to rounding to one decimal it may not always add up to 100%. The lines presenting the totals only refer to their respective subsection and should not be considered in the calculation of the columns totals.

**Attacks**

**X coordinates of the “elsewhere” fixations**

| Significant effects | df | χ² | *p* |
| --- | --- | --- | --- |
| Group | 2 | 5.44 | .066* |
| Time | 1 | 12.61 | <.001 |
| Time^2 | 1 | 11.03 | <.001 |
| Time^3 | 1 | 6.44 | .011 |

*In spite of a non-significant main effect, a simple effect was found between setters and controls: z = 2.35, *p* = .012.

**
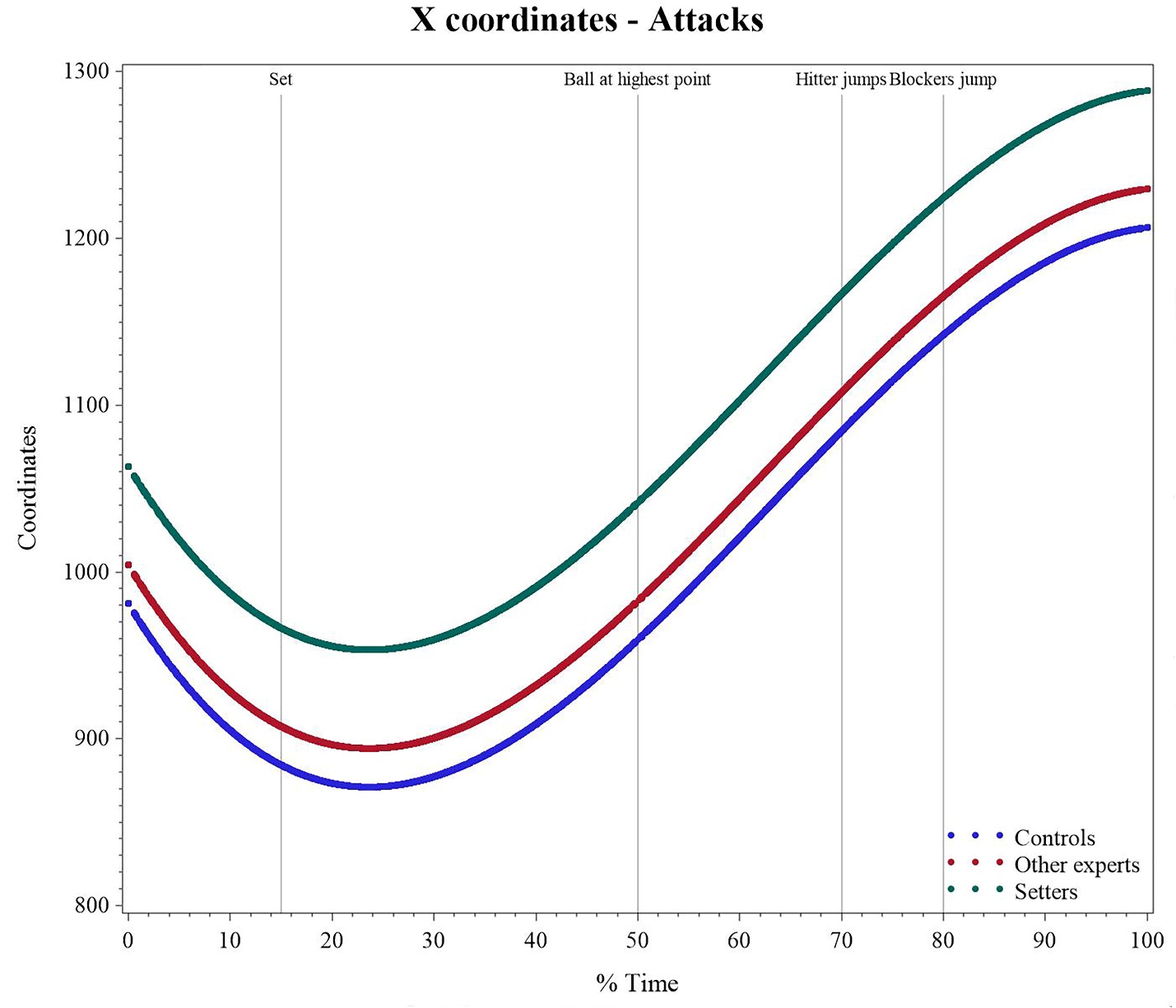
**

**Y coordinates of the “elsewhere” fixations**

| Significant effect | df | χ² | *p* |
| --- | --- | --- | --- |
| Group | 2 | 0.28 | .871 |
| Time | 1 | 26.59 | <.001 |
| Time*Group | 2 | 1.96 | .375 |
| Time^2 | 1 | 1.08 | .299 |
| Time^2*Group | 2 | 7.66 | .022* |
| Time^3 | 1 | 1.34 | .246 |
| Time^3*Group | 2 | 12.07 | .002* |
| Time^4 | 1 | 6.82 | .009 |
| Time^4*Group | 2 | 14.76 | .001* |

*No difference between groups at 10% of elapsed time (i.e., sets). At 30% of elapsed time (i.e., when ball is in motion towards hitter), only the difference between other players and controls was significant, z = 2.46, *p = .*014. At 50% of elapsed time (i.e., ball at highest point), differences were found between (1) setters and controls, z = 2.87, *p* = .004 and (2) controls and other experts, z = 3.07, *p = .*002. No differences were found from 70% of elapsed time onward (i.e., hitter jumps).

**
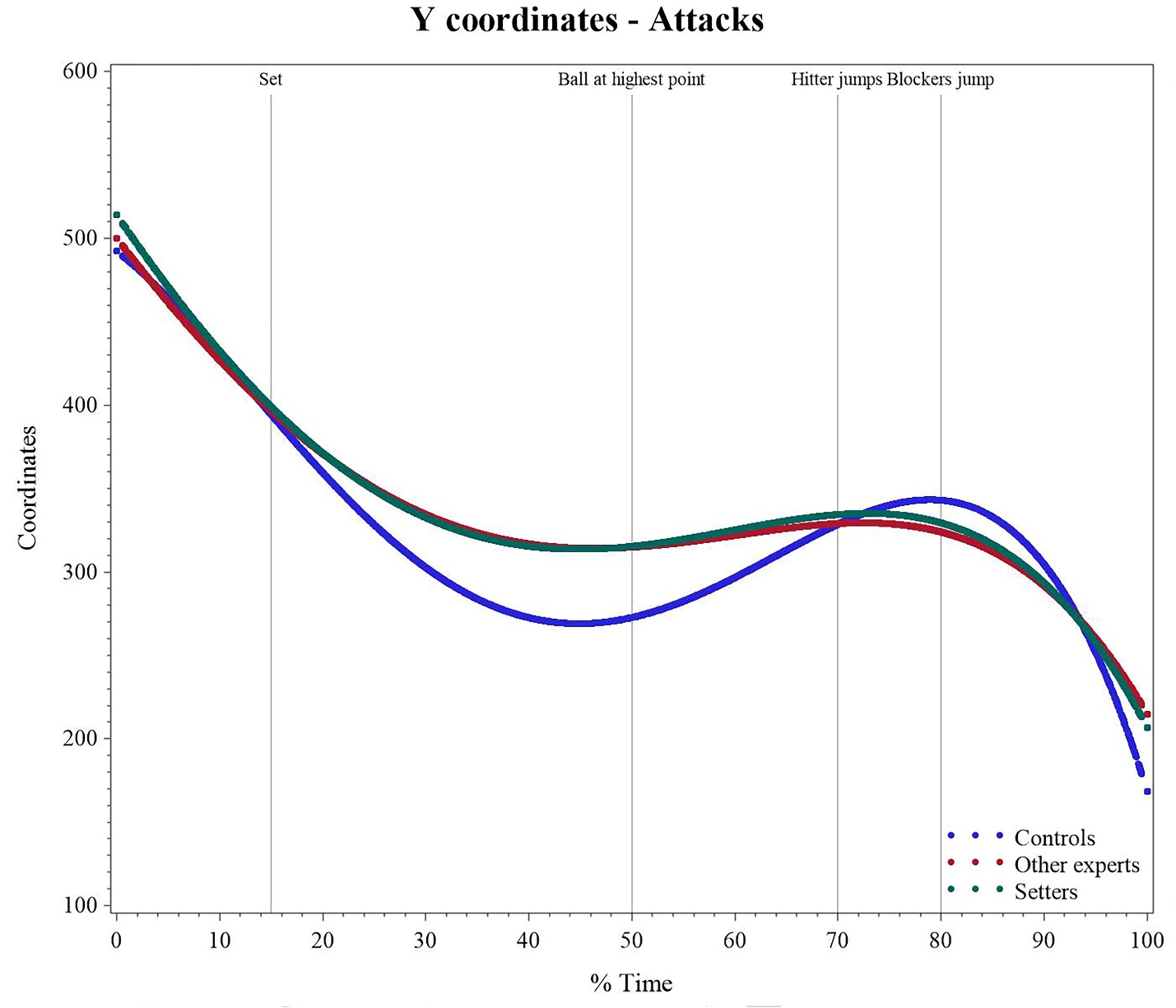
**

**Distribution of the location of the “elsewhere” fixations during the attack sequences**

|  | Moments (in % of viewing time) | | | | | | | | | | | | | | | | | | |
| --- | --- | --- | --- | --- | --- | --- | --- | --- | --- | --- | --- | --- | --- | --- | --- | --- | --- | --- | --- |
|  | 5 | | |  | 25 | | |  | 40 | | |  | 80 | | |  | 95 | | |
| Location | S | O-E | C |  | S | O-E | C |  | S | O-E | C |  | S | O-E | C |  | S | O-E | C |
| Attributed to an AOI |  |  |  |  |  |  |  |  |  |  |  |  |  |  |  |  |  |  |  |
| S’s upper body | 1.9 | 1.5 | 2.0 |  | 25.0 | 19.7 | 30.5 |  | 17.7 | 11.2 | 17.2 |  | 0.0 | 0.0 | 0.0 |  | 0.0 | 0.0 | 0.0 |
| S’s middle body | 5.8 | 6.0 | 8.0 |  | 0.9 | 0.7 | 1.2 |  | 0.0 | 0.0 | 0.0 |  | 0.0 | 0.0 | 0.0 |  | 0.0 | 0.0 | 0.0 |
| S’ lower body | 7.7 | 0.0 | 8.0 |  | 0.9 | 0.0 | 0.0 |  | 0.0 | 0.0 | 0.0 |  | 0.0 | 0.0 | 0.0 |  | 0.0 | 0.0 | 0.0 |
| O middle player | 34.6 | 38.8 | 24.0 |  | 14.8 | 19.7 | 4.9 |  | 4.4 | 10.1 | 3.5 |  | 3.4 | 0.0 | 3.9 |  | 0.0 | 0.7 | 1.1 |
| O blocker | 0.0 | 1.5 | 0.0 |  | 0.0 | 0.0 | 0.0 |  | 2.7 | 1.8 | 0.0 |  | 0.0 | 6.0 | 12.8 |  | 14.6 | 11.7 | 8.9 |
| Ball | 1.9 | 1.5 | 2.0 |  | 0.0 | 0.0 | 0.0 |  | 0.0 | 0.0 | 1.2 |  | 0.0 | 0.0 | 0.0 |  | 0.0 | 0.0 | 1.1 |
| Attacker | 0.0 | 0.0 | 0.0 |  | 0.0 | 0.0 | 0.0 |  | 7.1 | 4.1 | 1.2 |  | 73.9 | 69.5 | 64.1 |  | 69.9 | 70.8 | 66.7 |
| Total | 51.9 | 49.3 | 44.0 |  | 41.6 | 40.1 | 36.6 |  | 31.9 | 27.2 | 23.1 |  | 77.3 | 75.5 | 80.8 |  | 84.5 | 83.2 | 77.8 |
|  |  |  |  |  |  |  |  |  |  |  |  |  |  |  |  |  |  |  |  |
| Between two AOIs |  |  |  |  |  |  |  |  |  |  |  |  |  |  |  |  |  |  |  |
| S’s upper body – Ball | 0.0 | 0.0 | 4.0 |  | 0.0 | 0.0 | 0.0 |  | 1.8 | 0.0 | 2.3 |  | 0.0 | 0.0 | 0.0 |  | 0.0 | 0.0 | 0.0 |
| S’s middle body – Ball | 0.0 | 1.5 | 0.0 |  | 0.0 | 0.0 | 0.0 |  | 0.0 | 0.0 | 0.0 |  | 0.0 | 0.0 | 0.0 |  | 0.0 | 0.0 | 0.0 |
| S’ upper body – O blocker | 0.0 | 0.0 | 0.0 |  | 0.0 | 0.0 | 0.0 |  | 7.1 | 6.5 | 9.2 |  | 0.0 | 0.0 | 0.0 |  | 0.0 | 0.0 | 0.0 |
| O middle player – Ball | 0.0 | 1.5 | 0.0 |  | 0.0 | 0.0 | 0.0 |  | 0.0 | 0.0 | 0.0 |  | 0.0 | 0.0 | 0.0 |  | 0.0 | 0.0 | 0.0 |
| O middle player – O blocker | 1.9 | 0.0 | 0.0 |  | 1.9 | 2.9 | 2.4 |  | 0.9 | 0.0 | 4.6 |  | 0.0 | 0.0 | 0.0 |  | 0.0 | 0.0 | 0.0 |
| O middle player – S’ upper body | 3.9 | 1.5 | 0.0 |  | 45.4 | 35.8 | 42.7 |  | 25.7 | 25.4 | 33.3 |  | 0.0 | 0.0 | 0.0 |  | 0.0 | 0.0 | 0.0 |
| O middle player – S’ middle body | 3.9 | 10.5 | 8.0 |  | 0.0 | 0.0 | 0.0 |  | 0.9 | 0.6 | 0.0 |  | 0.0 | 0.0 | 0.0 |  | 0.0 | 0.0 | 0.0 |
| O middle player – S’ lower body | 0.0 | 4.5 | 6.0 |  | 0.0 | 0.0 | 0.0 |  | 0.0 | 0.0 | 0.0 |  | 0.0 | 0.0 | 0.0 |  | 0.0 | 0.0 | 0.0 |
| O middle player – S’ whole body | 19.2 | 22.4 | 20.0 |  | 2.8 | 10.2 | 9.8 |  | 0.0 | 0.6 | 1.2 |  | 0.0 | 0.0 | 0.0 |  | 0.0 | 0.0 | 0.0 |
| Attacker – O blocker | 0.0 | 0.0 | 0.0 |  | 0.0 | 0.0 | 0.0 |  | 5.3 | 15.4 | 3.5 |  | 20.5 | 23.2 | 16.7 |  | 12.6 | 13.0 | 18.9 |
| Attacker – O middle player | 0.0 | 0.0 | 0.0 |  | 0.0 | 0.0 | 0.0 |  | 0.0 | 1.2 | 0.0 |  | 0.0 | 0.0 | 0.0 |  | 0.0 | 0.7 | 0.0 |
| Total | 28.9 | 41.9 | 38.0 |  | 50.1 | 48.9 | 54.9 |  | 41.7 | 49.7 | 54.1 |  | 20.5 | 23.2 | 16.7 |  | 12.6 | 13.7 | 18.9 |
|  |  |  |  |  |  |  |  |  |  |  |  |  |  |  |  |  |  |  |  |
| In the vicinity of the main action | 5.8 | 4.5 | 6.0 |  | 5.6 | 8.8 | 6.1 |  | 0.0 | 0.0 | 0.0 |  | 0.0 | 0.0 | 0.0 |  | 0.0 | 0.0 | 0.0 |
|  |  |  |  |  |  |  |  |  |  |  |  |  |  |  |  |  |  |  |  |
| Out of the action | 13.5 | 6.0 | 10.0 |  | 2.8 | 2.2 | 2.4 |  | 26.6 | 23.1 | 23.0 |  | 2.3 | 1.3 | 2.6 |  | 2.9 | 3.3 | 3.3 |

*Notes.* S = Setters. O-E = Other experts. C = Controls. O = Opponent. AOI = Area of interest. The line regarding the setter’s whole body presents instances where the AOIs of parts of the setter’s body had to be fused and the third (or fourth) nearest AOI had to be verified. Each column totalizes 100% of the “elsewhere” fixations of each group at a given moment, but due to rounding to one decimal it may not always add up to 100%. The lines presenting the totals only refer to their respective subsection and should not be considered in the calculation of the columns totals.

**Blocks**

**X coordinates of the “elsewhere” fixations**

| Significant effect | df | χ² | *p* |
| --- | --- | --- | --- |
| Sequence | 8 | 72.83 | <.001 |
| Sequence*Time | 8 | 74.13 | <.001 |
| Group | 2 | 6.55 | .038* |
| Time | 1 | 55.55 | <.001 |
| Time^2 | 1 | 51.21 | <.001 |
| Time^3 | 1 | 40.36 | <.001 |
| Time^4 | 1 | 29.57 | <.001 |

*Difference between setters and controls: z = 2.75, *p* = .006. No difference between other players and controls: z = 1.50, *p* = .134, or between setters and other players: z = 1.42, *p* = .156.


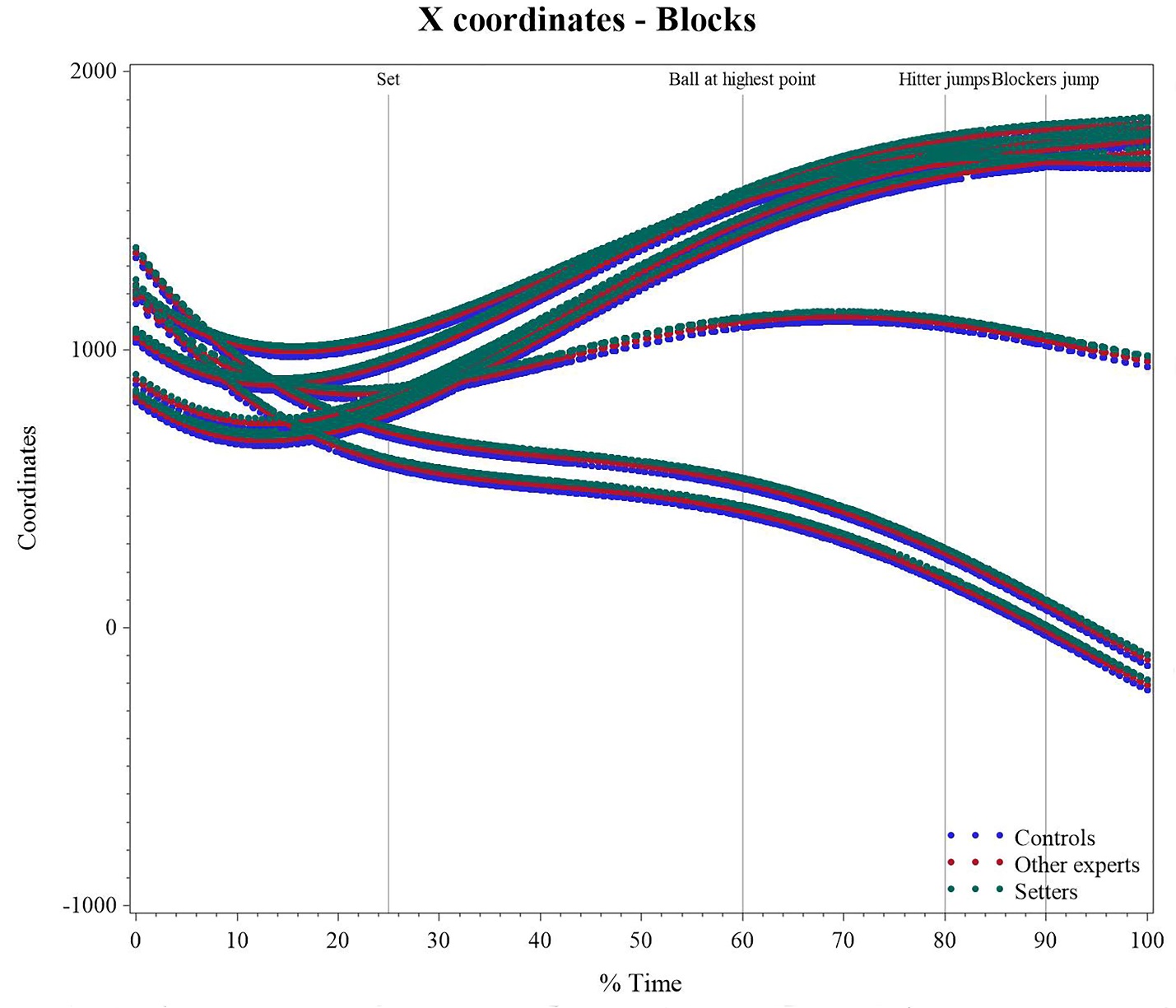


*Note.* The significant effects of (1) sequence and (2) the interaction between the sequence and time mean that the figure must contains three curves (one per group) per sequence to properly illustrate the evolution of the coordinates of the fixations.

**Y coordinates of the “elsewhere” fixations**

| Significant effect | df | χ² | *p* |
| --- | --- | --- | --- |
| Group | 2 | 4.35 | .113 |
| Time | 1 | 67.39 | <.001 |
| Time*Group | 2 | 8.51 | .014* |
| Time^2 | 1 | 64.52 | <.001 |
| Time^2*Group | 2 | 9.22 | .010* |
| Time^3 | 1 | 67.26 | <.001 |

*The only difference found was between controls and other experts at 50% of elapsed time, z = 2.01, *p* = .044 (i.e., ball close to highest point).


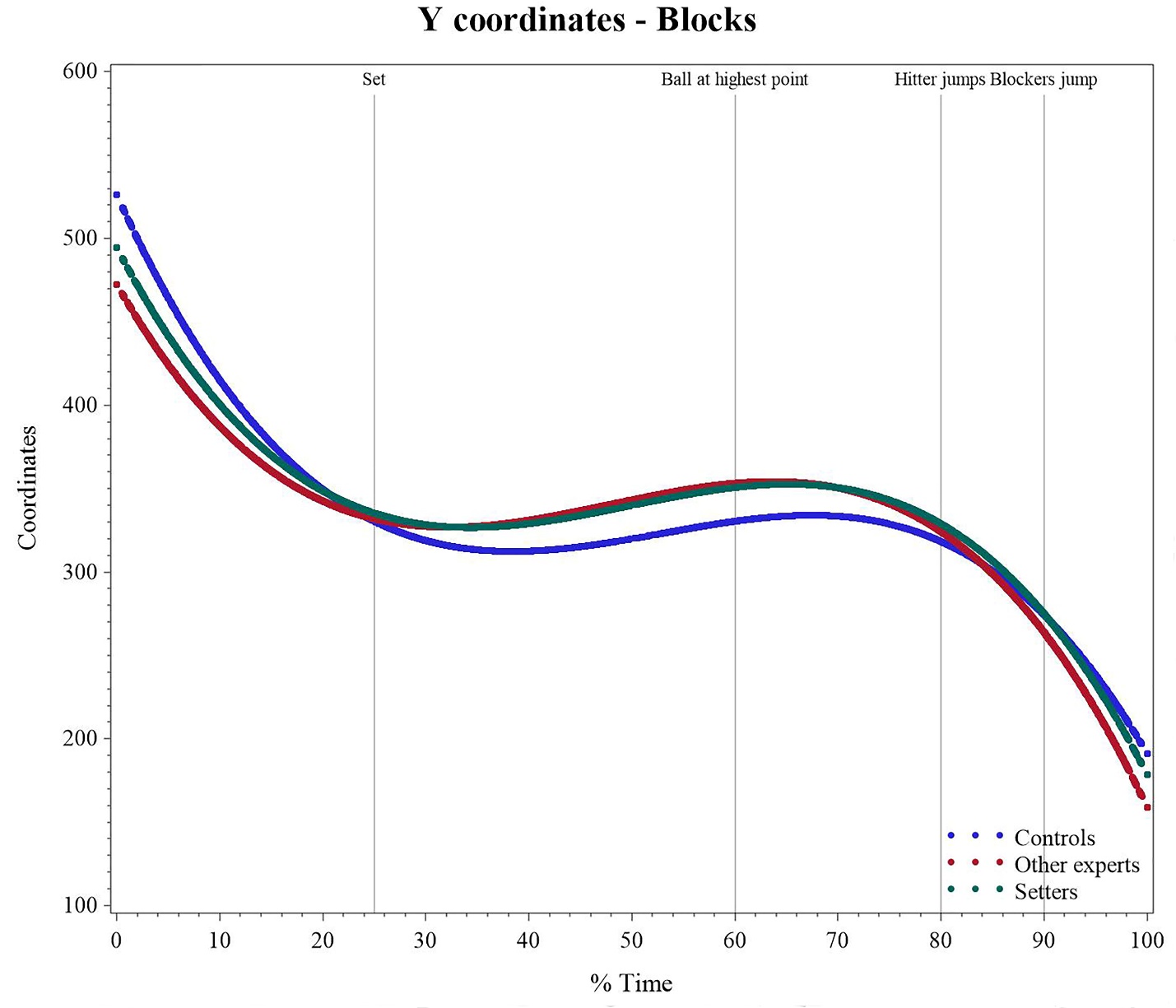


**Distribution of the location of the “elsewhere” fixations during the block sequences**

|  | Moments (in % of viewing time) | | | | | | | | | | | | | | |
| --- | --- | --- | --- | --- | --- | --- | --- | --- | --- | --- | --- | --- | --- | --- | --- |
|  | 15 | | |  | 35 | | |  | 70 | | |  | 95 | | |
| Location | S | O-E | C |  | S | O-E | C |  | S | O-E | C |  | S | O-E | C |
| Attributed to an AOI |  |  |  |  |  |  |  |  |  |  |  |  |  |  |  |
| S’s upper body | 7.0 | 9.8 | 9.2 |  | 3.0 | 7.3 | 2.3 |  | 0.0 | 0.0 | 0.0 |  | 0.0 | 0.0 | 0.0 |
| S’s middle body | 1.6 | 0.6 | 1.0 |  | 0.0 | 0.0 | 0.0 |  | 0.0 | 0.0 | 0.0 |  | 0.0 | 0.0 | 0.0 |
| S’s lower body | 0.0 | 0.6 | 0.0 |  | 0.0 | 0.0 | 0.0 |  | 0.0 | 0.0 | 0.0 |  | 0.0 | 0.0 | 0.0 |
| O middle player | 31.3 | 19.1 | 41.8 |  | 9.0 | 3.2 | 10.0 |  | 4.1 | 1.8 | 2.0 |  | 1.7 | 2.8 | 1.1 |
| O blocker | 0.8 | 0.0 | 0.0 |  | 2.4 | 0.0 | 0.0 |  | 12.2 | 16.8 | 9.8 |  | 21.4 | 22.0 | 22.6 |
| Ball | 0.0 | 1.7 | 0.0 |  | 0.0 | 0.0 | 0.0 |  | 0.0 | 0.0 | 1.0 |  | 0.0 | 2.3 | 1.1 |
| Attacker | 0.0 | 0.6 | 0.0 |  | 0.0 | 0.5 | 0.0 |  | 62.2 | 50.9 | 47.1 |  | 47.9 | 45.2 | 41.9 |
| Total | 40.7 | 32.4 | 52.0 |  | 14.4 | 11.0 | 12.3 |  | 78.5 | 69.5 | 59.9 |  | 71.0 | 72.3 | 66.7 |
|  |  |  |  |  |  |  |  |  |  |  |  |  |  |  |  |
| Between two AOIs |  |  |  |  |  |  |  |  |  |  |  |  |  |  |  |
| S’s upper body – Ball | 0.0 | 0.0 | 0.0 |  | 0.0 | 0.0 | 0.0 |  | 0.0 | 0.6 | 1.0 |  | 0.0 | 0.0 | 0.0 |
| S’ upper body – O blocker | 0.0 | 0.0 | 0.0 |  | 8.4 | 9.6 | 6.2 |  | 0.0 | 0.0 | 0.0 |  | 0.0 | 0.0 | 0.0 |
| O middle player – Ball | 2.3 | 2.9 | 0.0 |  | 0.0 | 0.0 | 0.0 |  | 0.0 | 0.0 | 1.0 |  | 0.9 | 0.6 | 2.2 |
| O middle player – O blocker | 0.8 | 1.2 | 0.0 |  | 7.8 | 8.2 | 8.5 |  | 0.0 | 1.2 | 6.9 |  | 0.0 | 0.6 | 0.0 |
| O middle player – S’ upper body | 24.2 | 29.5 | 23.5 |  | 45.8 | 35.9 | 46.9 |  | 0.0 | 1.2 | 1.0 |  | 0.0 | 0.0 | 0.0 |
| O middle player – S’ middle body | 0.8 | 6.9 | 1.0 |  | 0.0 | 0.9 | 0.0 |  | 0.0 | 0.0 | 0.0 |  | 0.0 | 0.0 | 0.0 |
| O middle player – S’ lower body | 0.8 | 0.0 | 3.1 |  | 0.0 | 0.0 | 0.0 |  | 0.0 | 0.0 | 0.0 |  | 0.0 | 0.0 | 0.0 |
| O middle player – S’ whole body | 13.3 | 12.1 | 13.3 |  | 1.2 | 3.6 | 0.8 |  | 0.0 | 0.0 | 0.0 |  | 0.0 | 0.0 | 0.0 |
| Attacker – O blocker | 0.0 | 0.0 | 0.0 |  | 6.6 | 8.6 | 1.5 |  | 17.4 | 22.8 | 17.7 |  | 12.8 | 16.4 | 15.1 |
| Attacker – O middle player | 6.3 | 1.7 | 1.0 |  | 0.0 | 0.0 | 0.0 |  | 2.0 | 1.2 | 3.9 |  | 7.7 | 6.2 | 8.6 |
| Attacker – S’s middle body | 0.0 | 1.2 | 1.0 |  | 0.0 | 0.0 | 0.0 |  | 0.0 | 0.0 | 0.0 |  | 1.7 | 0.0 | 0.0 |
| Attacker – Ball | 0.0 | 0.0 | 0.0 |  | 0.0 | 0.0 | 0.0 |  | 1.0 | 2.4 | 3.9 |  | 6.0 | 4.0 | 6.5 |
| Total | 48.5 | 55.5 | 42.9 |  | 69.8 | 66.8 | 63.9 |  | 20.4 | 29.4 | 35.4 |  | 29.1 | 27.8 | 32.4 |
|  |  |  |  |  |  |  |  |  |  |  |  |  |  |  |  |
| Out of the action | 10.9 | 11.6 | 5.1 |  | 15.7 | 22.3 | 23.9 |  | 1.0 | 1.2 | 4.9 |  | 0.0 | 0.0 | 1.1 |

*Notes.* S = Setters. O-E = Other experts. C = Controls. O = Opponent. AOI = Area of interest. The line regarding the setter’s whole body presents instances
where the AOIs of parts of the setter’s body had to be fused and the third (or fourth) nearest AOI had to be verified. Each column totalizes 100% of the
“elsewhere” fixations of each group at a given moment, but due to rounding to one decimal it may not always add up to 100%. The lines presenting the totals
only refer to their respective subsection and should not be considered in the calculation of the columns totals. No fixations were considered in the vicinity of
the action for block sequences.
